# Transmitted/Founder and Chronic Subtype C HIV-1 Use CD4 and CCR5 Receptors with Equal Efficiency and Are Not Inhibited by Blocking the Integrin α4β7

**DOI:** 10.1371/journal.ppat.1002686

**Published:** 2012-05-31

**Authors:** Nicholas F. Parrish, Craig B. Wilen, Lauren B. Banks, Shilpa S. Iyer, Jennifer M. Pfaff, Jesus F. Salazar-Gonzalez, Maria G. Salazar, Julie M. Decker, Erica H. Parrish, Anna Berg, Jennifer Hopper, Bhavna Hora, Amit Kumar, Tatenda Mahlokozera, Sally Yuan, Charl Coleman, Marion Vermeulen, Haitao Ding, Christina Ochsenbauer, John C. Tilton, Sallie R. Permar, John C. Kappes, Michael R. Betts, Michael P. Busch, Feng Gao, David Montefiori, Barton F. Haynes, George M. Shaw, Beatrice H. Hahn, Robert W. Doms

**Affiliations:** 1 Department of Microbiology, Perelman School of Medicine at the University of Pennsylvania, Philadelphia, Pennsylvania, United States of America; 2 Department of Medicine, Perelman School of Medicine at the University of Pennsylvania, Philadelphia, Pennsylvania, United States of America; 3 Department of Medicine, University of Alabama at Birmingham, Birmingham, Alabama, United States of America; 4 Duke Human Vaccine Institute, Duke University School of Medicine, Durham, North Carolina, United States of America; 5 Donation Testing Department, South African National Blood Service, Roodepoort, Gauteng, South Africa; 6 Department of General Medical Sciences, Center for Proteomics, Case Western Reserve University School of Medicine, Cleveland, Ohio, United States of America; 7 Department of Pediatrics, Duke University School of Medicine, Durham, North Carolina, United States of America; 8 Blood Systems Research Institute, San Francisco, California, United States of America; 9 Department of Medicine, Duke University School of Medicine, Durham, North Carolina, United States of America; 10 Department of Surgery, Duke University School of Medicine, Durham, North Carolina, United States of America; 11 Department of Immunology, Duke University School of Medicine, Durham, North Carolina, United States of America; University of Zurich, Switzerland

## Abstract

Sexual transmission of human immunodeficiency virus type 1 (HIV-1) most often results from productive infection by a single transmitted/founder (T/F) virus, indicating a stringent mucosal bottleneck. Understanding the viral traits that overcome this bottleneck could have important implications for HIV-1 vaccine design and other prevention strategies. Most T/F viruses use CCR5 to infect target cells and some encode envelope glycoproteins (Envs) that contain fewer potential N-linked glycosylation sites and shorter V1/V2 variable loops than Envs from chronic viruses. Moreover, it has been reported that the gp120 subunits of certain transmitted Envs bind to the gut-homing integrin α4β7, possibly enhancing virus entry and cell-to-cell spread. Here we sought to determine whether subtype C T/F viruses, which are responsible for the majority of new HIV-1 infections worldwide, share biological properties that increase their transmission fitness, including preferential α4β7 engagement. Using single genome amplification, we generated panels of both T/F (n = 20) and chronic (n = 20) Env constructs as well as full-length T/F (n = 6) and chronic (n = 4) infectious molecular clones (IMCs). We found that T/F and chronic control Envs were indistinguishable in the efficiency with which they used CD4 and CCR5. Both groups of Envs also exhibited the same CD4+ T cell subset tropism and showed similar sensitivity to neutralization by CD4 binding site (CD4bs) antibodies. Finally, saturating concentrations of anti-α4β7 antibodies failed to inhibit infection and replication of T/F as well as chronic control viruses, although the growth of the tissue culture-adapted strain SF162 was modestly impaired. These results indicate that the population bottleneck associated with mucosal HIV-1 acquisition is not due to the selection of T/F viruses that use α4β7, CD4 or CCR5 more efficiently.

## Author Summary

Most new HIV-1 infections worldwide are caused by the sexual transmission of subtype C viruses, which are prevalent in Asia and southern Africa. While chronically infected individuals harbor a genetically diverse set of viruses, most new infections are established by single variants, termed transmitted/founder (T/F) viruses. This raises the question whether certain viral variants have particular properties allowing them to more efficiently overcome the transmission bottleneck. Preferential binding of the viral envelope (Env) to the integrin α4β7 has been hypothesized as one important feature of transmitted viruses. Here, we compared Envs from subtype C viruses that were transmitted to those that were prevalent in chronic infections for efficiency in utilizing α4β7, CD4 and CCR5 for cell entry and replication. We found that transmitted and chronic Envs engaged CD4 and CCR5 with equal efficiency, and that blocking the interaction between Env and α4β7 failed to inhibit replication of T/F as well as control viruses. While the search for determinants of transmission fitness remains an important goal, preferential CD4, CCR5 or α4β7 interactions do not appear to represent distinguishing features of T/F viruses.

## Introduction

Mucosal transmission of HIV-1 is most often caused by a single variant from amongst the complex viral quasispecies in the infected donor [1]–[8]. After an eclipse phase of approximately two weeks during which virus is generally not detected in the blood, the progeny of this transmitted/founder (T/F) virus give rise to a productive systemic infection [9]–[15]. At a minimum, this significant population bottleneck selects for replication competent viruses, most of which use CCR5 as a coreceptor, since viruses that exclusively use CXCR4 are rarely transmitted [10], [16]. Whether other phenotypic traits are associated with enhanced mucosal transmission remains uncertain, though addressing this question is of importance because T/F viruses are the targets of vaccines, microbicides, and pre- and post-exposure prophylaxis.

Characterization of T/F virus properties is complicated by the challenges inherent in identifying acutely infected individuals, generating *bona fide* T/F molecular clones, procuring appropriate control viruses, obtaining sufficient numbers of samples to perform meaningful comparisons, and developing sufficiently sensitive *in vitro* assays to detect phenotypic differences that could impact transmission fitness *in vivo*. Almost all studies examining viral properties associated with mucosal transmission have focused on the viral envelope (Env) glycoprotein, most often in the context of viral pseudotypes [10], [17]–[20]. In addition, most initial studies examined viruses obtained weeks to months after infection from relatively few transmission events [21]–[23]. Given the rapidity with which HIV evolves in the face of immune pressures [24], “early” isolates could differ in important ways from true T/F viruses. Nonetheless, analyses of single genome amplification (SGA) derived T/F Env proteins and viruses have shown that mucosal transmission is associated with CD4+ T cell tropism and CCR5 use [10], [20], [25], [26] as well as a variety of signatures in the viral *env* gene [21], [27]–[32]. These include shorter variable loops, fewer potential N-linked glycosylation sites (PNGs) and, in some cases, enhanced sensitivity to neutralization by CD4 binding site (CD4bs) monoclonal antibodies (mAbs) [20]. More recently, it has been shown that the gp120 subunit of some Env glycoproteins can bind to, and signal through, the integrin α4β7 that is expressed on activated CD4+ T cells in the gut mucosa [33]–[35]. These findings have been taken to suggest that these interactions play an important role early in sexual transmission of HIV-1 [35], [36]. Specifically, it has been hypothesized that genetic signatures associated with transmission of certain subtype A and C viruses, including the absence of some PNGs in V1/V2 and C3/V4 regions, reflect selection for Envs that exhibit strong α4β7 binding and thus increased transmission fitness [35].

To explore the role of α4β7 interactions and other Env properties that might impact mucosal transmission, we employed SGA to generate a panel of T/F (n = 20) and chronic control (n = 20) Env constructs from geographically-matched individuals infected with subtype C viruses, the most prevalent HIV-1 lineage worldwide. To examine Env phenotypes in the context of replication competent viruses, we also produced full-length infectious molecular clones (IMCs) for six T/F and four chronic subtype C strains. Testing their biological activity in a variety of functional assays, we found no differences in the efficiency with which T/F and chronic Envs utilized CD4 or CCR5, mediated infection of primary CD4+ T cell subsets, or were neutralized by mAbs targeting the CD4bs. We confirmed that infection of α4β7-expressing CD4+ T cells by the prototypic subtype B strain HIV-1/SF162 could be partially inhibited by antibodies to α4β7 under some conditions as previously described [33], [34]. However, saturating concentrations of α4β7 antibodies had no inhibitory effect on infection of all-*trans* retinoic acid (atRA) stimulated CD4+ T cells from multiple donors by any of the T/F or chronic control viruses, even though most of their gp120 subunits are predicted to bind this integrin pair based on previously identified genetic signatures [35]. These findings indicate that the ability of some gp120 proteins to engage α4β7 may not be recapitulated by their native Env trimers on the surface of infectious particles, and thus suggests that interaction with this integrin pair is not critical for mucosal HIV-1 transmission.

## Results

### Generation of subtype C T/F and chronic Envs

Previous studies of T/F phenotypes focused almost exclusively on HIV-1 subtype B [10], [20], [37], [38]. To examine the extent to which these results are applicable to other subtypes, we focused in this study on the transmission properties of HIV-1 subtype C. To assess viral entry, we assembled a panel of 20 T/F Env clones, six of which have previously been described [39]. The remaining 14 clones were derived from 13 acutely infected individuals from South Africa and Zambia (8 males, 5 females) - nine of whom were sampled during the earliest stages of viral infection (Fiebig stages I and II [40]; Table 1). Plasma viral RNA was extracted, subjected to SGA and direct amplicon sequencing, and used to infer the T/F *env* sequences as previously described [12]. Consistent with earlier findings, infection was established by one or a limited number of viral variants. Of the 18 acutely infected individuals included in this panel, 14 acquired a single variant, while three others were infected with two variants and one was infected by four variants (Table 1 and Figure S1).

**Table 1:** Origin of T/F and chronic HIV-1 *env* clones.

Env type	Subject	Env clone designation	Country	Risk factor^a^	Sex	Viral load (RNA copies/ml)	Fiebig stage^b^	Number of SGA sequences	Number of T/F variants	Reference	Cohort^g^
T/F	20258279	20258279-V2_3A5^d^	S. Africa	SPD	F	281,838	IV	41	4	this study	SANBS
	20258279	20258279-V4_3D10^d^	S. Africa	SPD	F	281,838	IV	41	4	this study	SANBS
	2833264	2833264_3G11	S. Africa	SPD	M	234,423	I/II	13	1	this study	SANBS
	21197826	21197826-V1_3A1	S. Africa	SPD	F	343,923	I/II	13	2	this study	SANBS
	21283649	21283649_3E8	S. Africa	SPD	M	3,180	I/II	27	1	this study	SANBS
	20927783	20927783_3E2	S. Africa	SPD	F	1,886	I/II	11	1	this study	SANBS
	1245045	1245045_3C7	S. Africa	SPD	M	234,068	I/II	10	1	this study	SANBS
	19157834	19157834-V1_3C3	S. Africa	SPD	M	275,423	I/II	36	2	this study	SANBS
	2935054	2935054_3A3	S. Africa	SPD	M	>10,000,000	I/II	18	1	this study	SANBS
	ZM246F^c^	ZM246F_C1G^c^	Zambia	HSX	F	10,013,800	II	41	1	9, 25, 38	ZEHRP
	ZM247F	ZM247Fv1.Rev-d	Zambia	HSX	F	10,823,500	II	44	2	9, 25, 38	ZEHRP
	ZM247F	ZM247Fv2.fs^d^	Zambia	HSX	F	10,823,500	II	44	2	9, 25, 38	ZEHRP
	ZM249M^c^	ZM249M-B10^c^	Zambia	HSX	M	>2,000,000	IV	49	1	9, 25	ZEHRP
	704809221	704809221.1B3	S. Africa	HSX	M	>750,000	I/II	28	1	12, 38	CHAVI
	703010054	703010054.2A2	Malawi	HSX	M	13,936	V	27	1	12, 38	CHAVI
	703010217	703010217.B6	Malawi	HSX	F	102,602	V/VI	25	1	12 38	CHAVI
	706010018	706010018.2E3	S. Africa	HSX	F	93,700	VI	23	1	12	CHAVI
	704010042^c^	704010042.2E5	S. Africa	HSX	M	181,000	IV	42	1	12	CHAVI
	705010198	705010198.tf	S. Africa	HSX	M	14,950,000	I/II	10	1	in prep^f^	CHAVI
	705010185	705010185.tf	S. Africa	HSX	F	14,800	I/II	10	1	in prep^f^	CHAVI
Chronic	704010330	704010330.G5h	S. Africa	HSX	M	46,100	n/a	26	n/a	this study	CHAVI
	704010207	704010207.D11	S. Africa	HSX	F	15,400	n/a	26	n/a	this study	CHAVI
	702010141	702010141.synR1	Malawi	HSX	F	151,282	n/a	39	n/a	this study	CHAVI
	703010180	703010180.A3^e^	Malawi	HSX	F	105,430	n/a	15	n/a	this study	CHAVI
	702010432^c^	702010432.synR1^c^	Malawi	HSX	M	40,570	n/a	30	n/a	this study	CHAVI
	703010167	703010167.synR1	Malawi	HSX	F	73,505	n/a	30	n/a	this study	CHAVI
	ZM414	ZM414.1^d^	Zambia	HSX	F	213,600	n/a	41	n/a	40	TDRC
	ZM414	ZM414.20^d^	Zambia	HSX	F	213,600	n/a	41	n/a	40	TDRC
	707010457^c^	707010457.synR1^c^	Tanzania	HSX	F	234,671	n/a	20	n/a	this study	CHAVI
	705010534^c^	705010534.synR1^c^	S. Africa	HSX	F	63,300	n/a	36	n/a	this study	CHAVI
	704010499	704010499.H1	S. Africa	HSX	F	15,200	n/a	21	n/a	this study	CHAVI
	704010461	704010461.A7h	S. Africa	HSX	F	22,900	n/a	7	n/a	this study	CHAVI
	704010028	704010028.F6	S. Africa	HSX	F	9,220	n/a	20	n/a	this study	CHAVI
	703010269	703010269.synR1	Malawi	HSX	F	30,434	n/a	30	n/a	this study	CHAVI
	704010273	704010273.E5^e^	S. Africa	HSX	F	25,700	n/a	24	n/a	this study	CHAVI
	709013902	3902.bmG14	Malawi	HSX	F	19,900	n/a	20	n/a	41, 43	CHAVI
	709014707	4707.E1	Malawi	HSX	F	83,400	n/a	22	n/a	41, 43	CHAVI
	709014403	4403.A18^d^	Malawi	HSX	F	100,892	n/a	38	n/a	41, 43	CHAVI
	709014403	4403.D1^d^	Malawi	HSX	F	100,892	n/a	38	n/a	41, 43	CHAVI
	709014403	4403.bmB6^d^	Malawi	HSX	F	100,892	n/a	38	n/a	41, 43	CHAVI

a SPD = source plasma donors who denied having sex for money or with multiple partners, homosexual activity, injection drug use, or receiving a blood transfusion or a tattoo in the preceding six months; HSX = heterosexual exposure.

b defined in [40]; n/a = not applicable for subjects enrolled in chronic cohorts.

c IMC from subject also tested, see Table 2.

d multiple Env clones from single subject.

e Env differs from “rake” consensus by a single amino acid.

f Kappes et al., manuscript in preparation.

g SANBS = South African National Blood Service; ZEHRP = Zambia-Emory HIV Research Project; CHAVI = Center for HIV/AIDS Vaccine Immunology; TDRC = Tropical Diseases Research Centre.

**Figure 1:**
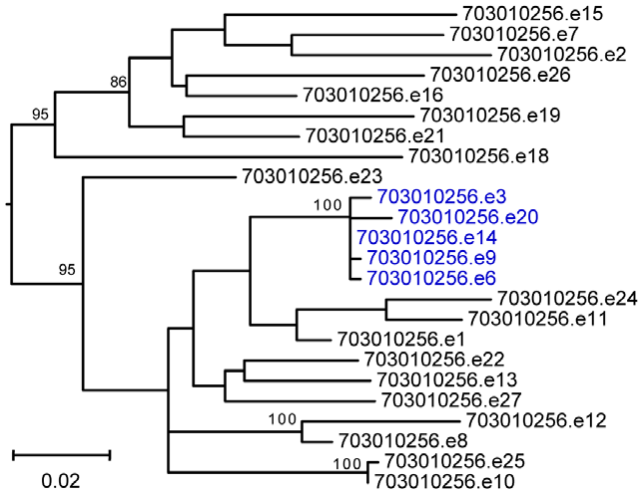
Clonal virus expansion in a chronically infected subject. The phylogenetic relationships of SGA-derived 3′ half genome sequences depicting the quasispecies complexity in a chronically infected subject are shown. Sequences highlighted in blue are members of a recently expanded lineage, the consensus sequence of which approximates their most recent common ancestor. The tree was constructed using maximum likelihood methods [82]. Nodes with bootstrap support of greater than 80% are labeled (the scale bar represents 0.02 nucleotide substitutions per site).

To generate an appropriate control group, we obtained 20 Env clones from individuals chronically infected with subtype C viruses (Table 1). Seven of these have previously been described [41], [42]. The remaining 13 were generated from chronically infected individuals (11 females, 2 males) enrolled in the CHAVI 001 cohort [43]. While T/F Envs were derived from individuals of both sexes (10 males; 10 females), chronic Envs were predominantly derived from female subjects (2 males; 18 females). To increase the probability of identifying functional *env* genes, we used SGA to generate up to 42 *env* gene sequences for each chronically infected individual (Figures S2 and S3). We then constructed phylogenetic trees to identify viruses that had undergone a recent clonal expansion as evidenced by clusters or “rakes” of closely related sequences (Figures 1, S2 and S3). We reasoned that the common ancestor of such clonally expanded “rakes” would be more likely to encode a fully functional *env* gene than a sequence chosen at random from the quasispecies. To approximate this ancestor, we cloned *env* amplicons whose sequences were either identical to the consensus sequence of the corresponding rake (n = 5) or encoded an Env that differed in a single amino acid residue (n = 2). For subjects from whom none of the *env* amplicons met these criteria, the rake consensus sequence was inferred and chemically synthesized (n = 6). This same approach had also been employed to generate the previously reported chronic Env constructs [41], [44]. Thus, all 20 chronic control Envs used in this study were derived from clonally expanded viruses.

### Functional analysis of subtype C T/F and chronic Envs

Virus pseudotypes containing a luciferase reporter gene and bearing one of the T/F or chronic control Envs were produced in human 293T cells and then diluted serially on NP2/CD4/CCR5 and NP2/CD4/CXCR4 cells to assess coreceptor usage and to determine the linear range of the assay. All viral pseudotypes were functional, leading to infection of NP2/CD4/CCR5 cells at least 100-fold above Env-negative particles. In contrast, none of the T/F Envs and only one chronic control Env (4707.E1) mediated entry into NP2/CD4/CXCR4 cells at levels 10-fold above background. However, this Env did not mediate entry of GFP-encoding pseudoviruses into primary CD4+ T cells in the presence of the CCR5 antagonist maraviroc (data not shown). Thus, the small amount of CXCR4-dependent infection seen in NP2/CD4/CXCR4 cells is likely due to the over-expression of this coreceptor and does not reflect CXCR4 use on primary cells. Importantly, all T/F and chronic Env constructs were functional, thus validating our methods to correctly infer T/F as well as clonally expanded chronic control viruses.

**Figure 2:**
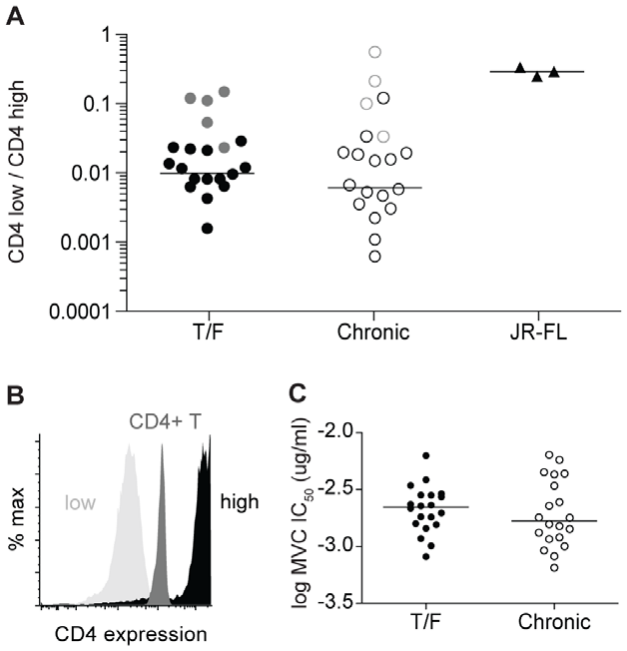
T/F and chronic Envs utilize CD4 and CCR5 with similar efficiency. (**A**) Efficiency of CD4 usage by T/F and chronic Env pseudoviruses. Luciferase-encoding pseudoviruses were used to infect affinofile cells with maximally-induced CCR5 expression and either low or high CD4 expression. Infection of CD4-low cells was measured based on luciferase activity and normalized to infection of CD4-high cells (*y*-axis). Data shown are the mean value from three independent experiments for all T/F and chronic Envs, each tested in triplicate; for JR-FL the mean of each of three experiments is plotted. Grey circles indicate poorly infectious Envs for which the CD4 use efficiency is falsely elevated because infection of CD4-low cells was near background, as described in the methods. The bar represents the median value of each group with these Envs excluded. There was no significant difference in the CD4-use efficiency between T/F and chronic envelopes (Mann-Whitney; *p* = 0.35). (**B**) CD4 expression levels from a representative flow cytometry experiment depicting CD4 expression on minimally-induced Affinofile cells (low), fully-induced Affinofile cells (high), and primary human CD4+ T cells (CD4+ T). (**C**) Sensitivity of T/F and chronic Envs to inhibition by maraviroc (MVC). Viral pseudotypes were used to infect NP2/CD4/CCR5 cells in the presence of serial three-fold dilutions of the CCR5 antagonist maraviroc. The concentration required to reduce infection by 50% relative to no drug control (IC_50_) was calculated for each pseudotype. Log MVC IC_50_ values are shown in the y-axis. The bar represents the median value of each group. Higher IC_50_ values indicate that more drug is required to prevent infection corresponding to more efficient CCR5 usage, whereas lower IC_50_ values correspond to less efficient CCR5 usage. No significant difference in IC_50_ values was found between T/F and chronic envelope pseudotypes (Mann-Whitney; *p* = 0.46).

Enhanced utilization of CD4 and CCR5 could influence virus transmission since changes in CD4 and CCR5 expression have been shown to impact infection by different HIV-1 strains [45]–[49]. A previous study of subtype B T/F and chronic viral Envs did not reveal differences in their utilization of CCR5 [20]. To determine the efficiency with which the newly derived subtype C T/F Envs utilized CD4 relative to the chronic Env controls, we compared their ability to infect affinofile cells, a 293T cell line that expresses CD4 and CCR5 under independently inducible promoters [50] (Figure 2). We induced CCR5 to maximal levels and induced CD4 to high or low expression levels (relative to primary human CD4+ T cells; Figure 2B) prior to infection. Infection levels of each pseudovirus in the CD4-low cells were then expressed relative to the values obtained in the CD4-high cells (Figure 2A). The macrophage-tropic JR-FL Env, which is known to mediate efficient entry into cells expressing low levels of CD4 [51], [52], was used as a control. Using this system, we found that virus pseudotypes expressing T/F and clonally expanded chronic Envs utilized CD4 with similar efficiency, while CD4 use by JR-FL was 10-fold more efficient than most of the other pseudoviruses (Figure 2A). These results demonstrated that the affinofile system is sufficiently sensitive to detect differences in CD4 utilization amongst different virus strains. Additionally, the results confirmed earlier studies of subtype B and C viruses, which indicated that the ability to use limiting levels of CD4 is not a major determinant of transmission fitness [18], [37].

To assess the efficiency of CCR5 use, we infected NP2/CD4/CCR5 cells in the presence of increasing concentrations of the CCR5 antagonist maraviroc and measured the IC_50_ value for each virus. We chose this approach over the use of affinofile cells since we have found that CCR5 expression levels cannot be controlled with sufficient precision at intermediate concentrations of the inducing reagent. Moreover, maraviroc titration should impact CCR5 availability to the same degree on all cells in the population. Therefore, maraviroc sensitivity is a surrogate for the efficiency of CCR5 use, provided that none of the Envs tested can use CCR5 when it is bound to maraviroc [53], [54]. This was true of our Env panel; all T/F and chronic Envs examined were equally sensitive to saturating concentrations of maraviroc with maximal percent inhibitions of >95%. Additionally, T/F and chronic Envs exhibited similar maraviroc IC_50_ values (median T/F = 2.22 nM; chronic = 1.67 nM; p = 0.45). Thus, enhanced CCR5 utilization efficiency does not account for the profound transmission bottleneck of both subtype B [20] and C (Figure 2) [18] infections.

### CD4+ T cell subset tropism of subtype C T/F and chronic Envs

**Figure 3:**
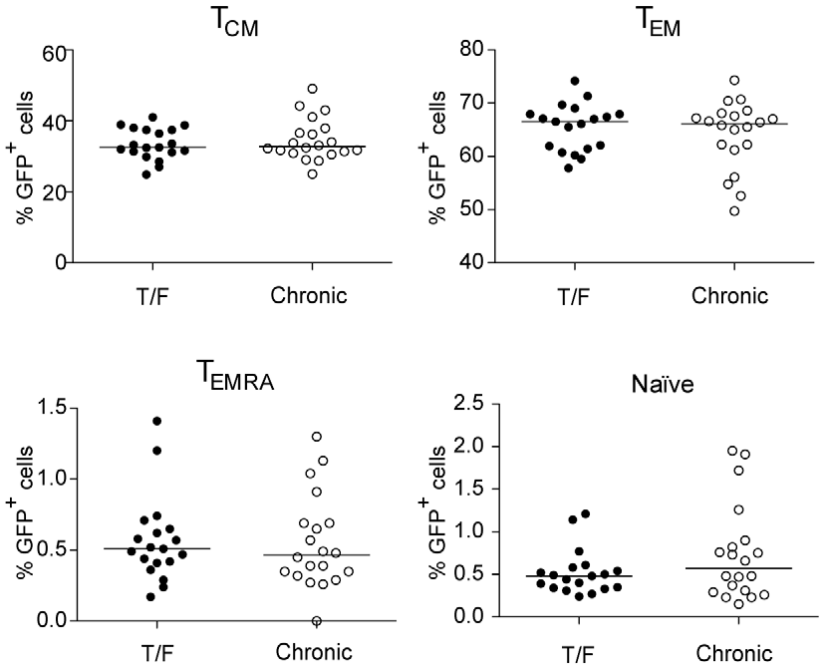
T/F and chronic Env pseudotypes enter primary CD4+ T cell subsets with similar efficiency. Primary CD4+ T cells were infected with GFP encoding Env pseudotypes to assess the ability of T/F and chronic Envs to mediate entry into different CD4+ T cell subsets. Three days post-infection, cells were stained for viability as well as CD3, CD4, CD45RO and CCR7 expression [20]. In three independent experiments, cells from different donors cells were analyzed by flow cytometry and GFP+ cells were back-gated onto memory markers to evaluate differential infection of subsets; the average percent of infected cells falling into each subset for each Env is plotted on the *y*-axis. Cells were classified as central memory (T_CM_: CCR7+CD45RO+), effector memory (T_EM_: CCR7−CD45RO+), effector memory RA (T_EMRA_: CCR7−CD45RO−), and naïve (CCR7+CD45RO−). While T_EM_ cells permitted the most efficient infection by Env pseudoviruses, there was no significant difference in infection efficiency between T/F and chronic Envs in any of the subsets. The bar represents the median percentage of cells of each subset infected by T/F or chronic pseudoviruses.

CD4+ T cell subsets have different activation and coreceptor expression levels, and thus may be differentially susceptible to infection by T/F versus chronic control viruses [55]–[57]. Effector memory (T_EM_) and effector memory RA^+^ (T_EMRA_) cells predominate in mucosal effector sites where the transmission bottleneck likely occurs, while central memory (T_CM_) and naïve cells are more common in lymph nodes [58], [59]. Therefore, an enhanced ability to infect T_EM_ and T_EMRA_ cells could be linked to enhanced mucosal transmission. To explore this, we infected primary CD4+ T cells with GFP-expressing pseudoviruses and then stained for CCR7 and CD45RO to define naïve (CCR7+CD45RO−), T_CM_ (CCR7+CD45RO+), T_EM_ (CCR7−CD45RO+), and T_EMRA_ (CCR7−CD45RO−) cells. As shown in Figure 3, we saw no differences in the abilities of subtype C T/F and chronic control Envs to mediate entry into these CD4+ T cell subsets. Similar to our observations for subtype B T/F and control Envs [20], most infected cells were T_EM_, but T/F Envs showed no preference for this cell type relative to chronic control Envs.

### Neutralization sensitivity of subtype C T/F and chronic Envs

**Figure 4:**
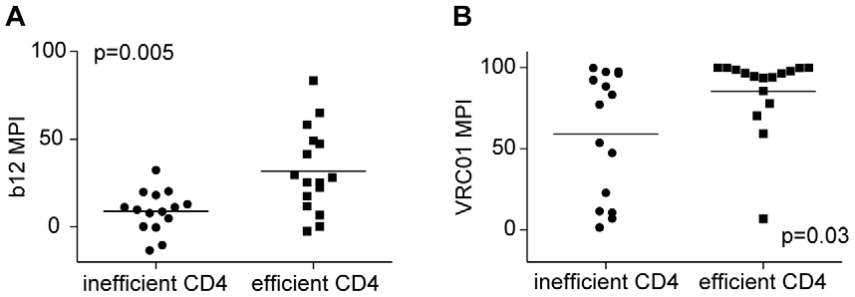
CD4-use efficiency correlates with CD4 binding site neutralization sensitivity. The *y*-axis shows sensitivity to CD4bs mAbs b12 (**A**) and VRC01 (**B**) as measured by the maximal percent inhibition (MPI) using 10 µg/ml of each mAb. Envs were divided according to their CD4-use efficiency; those that used CD4 efficiently were more sensitive to CD4bs antibodies than Envs that used CD4 inefficiently. The bar represents the median MPI value and p-values are from two-tailed Mann-Whitney tests.

We recently reported that subtype B T/F Envs were more sensitive than chronic Envs to the CD4bs mAbs b12 and VRC01, and that this was attributable to increased binding of these antibodies to the native Env trimer [20]. To determine whether this was also true for subtype C, we performed neutralization assays with the same antibodies. No significant differences in neutralization sensitivity to b12, VRC01, PG9 or PG16 were observed for T/F and chronic control Envs (Figure S4). As expected, VRC01 generally neutralized subtype C Envs more potently than b12 [60]. Using 10 µg/ml of b12, only five Envs were inhibited by 50%, and the most sensitive Env was inhibited by 83%. Using the same concentration of VRC01, 25 Envs were inhibited by 83%, and 16 Envs were inhibited by 95%. Nonetheless, we noted a relationship between the sensitivity to both VRC01 and b12 and the efficiency of CD4 use. When Envs were divided into those that used CD4 most efficiently (top 50% regardless of whether they represented T/F or chronic controls) and those that used CD4 least efficiently, the Envs that used CD4 efficiently were more sensitive to CD4bs (Figure 4). In contrast, no relationship between CD4-use efficiency and neutralization sensitivity was observed for PG9, PG16, or purified immunoglobulin pooled from five individuals infected with subtype C HIV-1 (data not shown). Thus, subtype C T/F and chronic Envs used CD4 with similar efficiency, and there was a correlation between CD4 utilization and sensitivity to neutralization by CD4bs mAbs.

### Effect of α4β7 blockade on infection by Env pseudotypes

The gut-homing integrin α4β7 is expressed on activated CD4+ T cells in the gut [61], [62] and vaginal mucosa [63] and has been shown to bind the gp120 proteins from several recently transmitted subtype A and subtype C viruses [35]. In contrast, gp120 proteins from chronic viruses appear to bind α4β7 only rarely, although the subtype B HIV-1/SF162 strain is a notable exception [35]. Only a few studies have examined the effect of Env-α4β7 interactions on virus replication [33], [34], [64]. It has been shown that mAbs specific for α4β7 partially and transiently inhibit infection of α4β7-positive CD4+ T cells by HIV-1/SF162 at low inocula. Based on these studies, it has been suggested that engagement of α4β7 may enhance HIV-1 infection, especially in the context of mucosal transmission [35], [36].

If the ability of gp120 proteins to bind α4β7 is recapitulated by Env molecules present on virus particles, we reasoned that α4β7 engagement should enhance virus entry, especially at low multiplicities of infection, since binding to the cell surface is a rate-limiting step of virus infection *in vitro* [65], [66]. If so, then saturating levels of mAbs to α4β7 should suppress virus infection, as has been shown for the subtype B virus strain HIV-1/SF162 [33], [34]. To investigate this, we used a protocol previously developed by Arthos and colleagues in which human CD4+ T cells were stimulated with IL-2 (20 IU/ml), anti-CD3 (1.5 µg/ml) and atRA (10 nM). Under these growth conditions, α4β7 expression was enhanced and detected on 15–65% (median 32%) of CD4+ T cells, predominantly on effector memory cells, from six different donors (data not shown). One donor was non-responsive to atRA and expressed α4β7 on only five to six percent of CD4+ T cells at day six, so these cells were not used for subsequent experiments.

**Figure 5:**
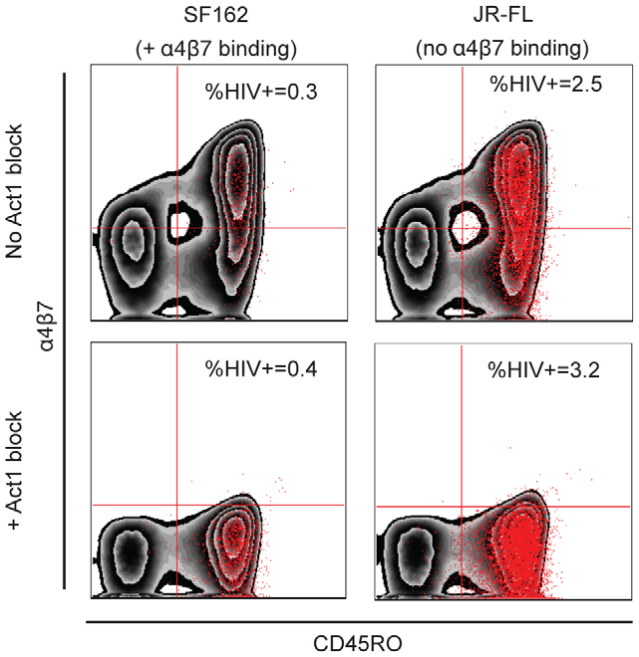
Blocking α4β7 enhances pseudovirus infection. Infection of primary CD4+ T cells by Env pseudoviruses is depicted in the presence and absence of Act1 to block α4β7. atRA-treated cells were infected with GFP-expressing pseudotypes containing SF162 and JR-FL Envs whose gp120 proteins do and do not bind α4β7, respectively, in the absence (top two panels) or presence of saturating amounts of the α4β7 specific mAb Act1 (bottom two panels). Infected cells were detected by GFP expression (red overlay). The presence of Act1 resulted in slightly increased infection levels of both pseudotypes. To confirm that saturating levels of Act1 were used, cells were stained with fluorescently labeled Act1 before analysis. The near absence of cells in the upper quadrants of the lower panels shows that binding of labeled Act1 antibody was blocked by unlabeled antibody added before virus infection.

We next titrated two commercially available α4β7 mAbs, Act1 (specific for the α4β7 heterodimer) and 2B4 (specific for α4), both of which have been shown to inhibit gp120 binding and to suppress infection of atRA-treated CD4+ T cells by the laboratory adapted HIV-1/SF162 strain, using concentrations previously reported to be saturating for Act1 [33]. To determine if our infection and inhibition conditions were sufficiently sensitive, we used GFP reporter-expressing SF162 Env-containing pseudovirus as the positive control. Pseudovirus expressing the JR-FL Env served as the negative control, since the JR-FL gp120 does not bind α4β7 [35]. We failed to detect any inhibition of infection by either pseudovirus at a broad range of inocula using saturating concentrations of Act1 (Figure 5). In fact, Act1 treatment enhanced infection of SF162, JR-FL and VSV-G pseudoviruses by approximately 30% in cells from two different donors. However, gp120-α4β7 binding has recently been described to be critically dependent on Env glycosylation, with high mannose carbohydrates enhancing and complex glycans reducing α4β7 interactions [35]. We therefore reasoned that viruses derived from primary CD4+ T cells would be physiologically more relevant, since these cells produce Env proteins with predominantly high-mannose carbohydrates that support α4β7 binding [67].

### Effect of α4β7 blockade on replication of subtype C T/F and chronic IMCs

**Figure 6:**
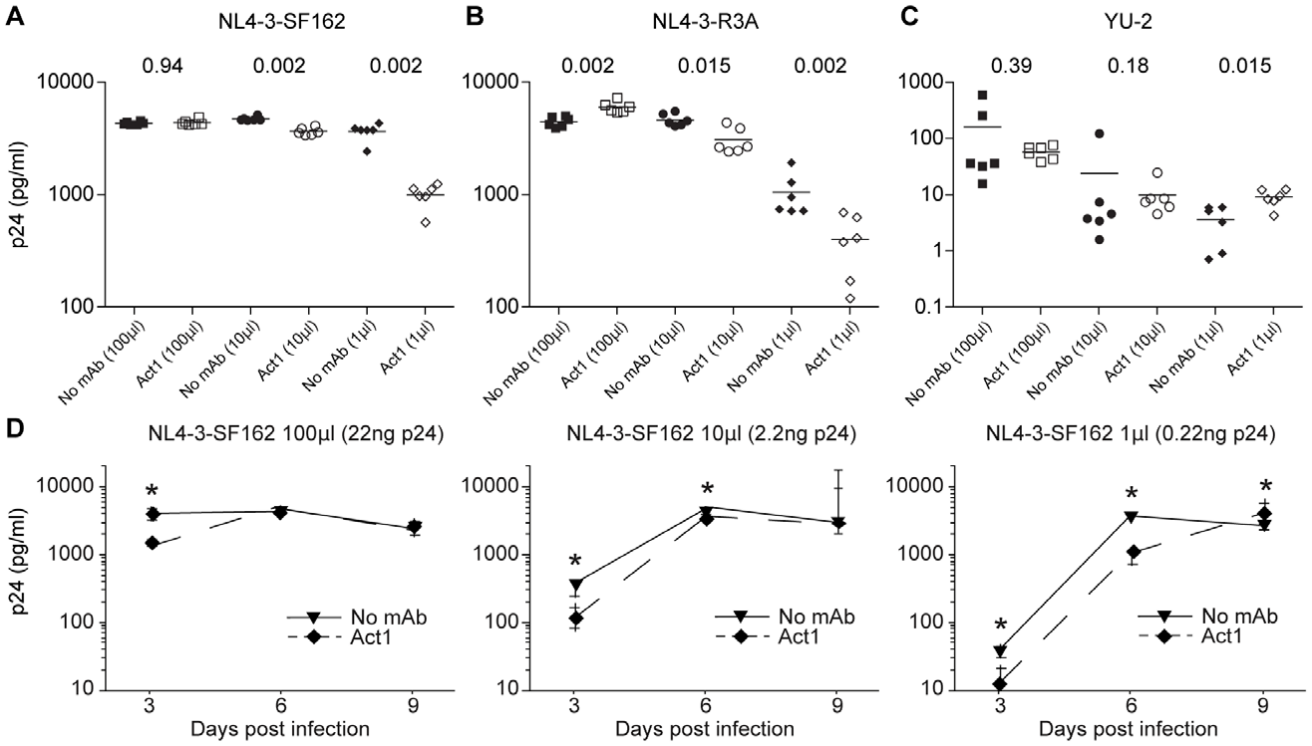
Blocking α4β7 inhibits replication of NL4-3-SF162 and NL4-3-R3A but not YU-2. CD4+ T cells with or without Act1 pre-treatment were infected at three different multiplicities using CD4+ T cell derived virus stock (1 µl, 10 µl and 100 µl) to initiate a spreading infection. Infections were performed in six replicate wells, each of which was sampled at days three, six and nine. (**A-C**) Virus production at day six as measured by p24 content in culture supernatants is shown on the y-axis for each of six replicate wells from one of three independent experiments; uncorrected Mann-Whitney *p* values are shown for comparisons of no antibody (solid symbols) versus Act1-treated (open symbols) replicate wells (bar = mean). (**D**) Replication kinetics are shown for NL4-3-SF162 (mean of replicates ± SEM is presented) at three different multiplicities of infection. Inhibition of infection was transient and greatest at six days post infection at the lowest viral input; uncorrected Mann-Whitney p-values less than 0.05 comparing no mAb to Act1 are marked by asterisks.

To examine the impact of α4β7 blockade on the infectivity and growth kinetics of replication competent viruses, we generated full-length subtype C IMCs representing T/F (n = 6) and chronic control (n = 4) viruses (Table 2 and Figure S3). All but three of these had the LDI/V tripeptide motif in the V2 loop, which has been shown to play a key role in gp120-α4β7 binding (Figure S5) [33]–[35]. Moreover, the number of N-linked glycosylation sites in the V1/V2 region of these IMCs (range from 3 to 8) was comparable to that in gp120 proteins known to interact with α4β7 (range 3 to 9 for the strains SF162, 205F, QA203, and CAP88 [35]). Finally, all replication competent virus stocks were produced in primary human CD4+ T cells to ensure physiologically relevant Env glycosylation, processing and virion incorporation. Using these reagents, we infected atRA-treated primary CD4+ T cells with each virus strain, using a wide range (100-fold) of inocula in the presence of saturating concentrations of Act1. In three independent experiments, we found that Act1 consistently inhibited replication of an SF162 Env-containing molecular clone (NL4-3-SF162, gift from J. Arthos) at six days post-infection. This inhibition was greatest at the lowest multiplicity of infection (Figure 6 A and D). We also observed significant Act1-mediated inhibition of an NL4-3 construct that encoded the subtype B Env R3A [68], but again this was seen only at the two lowest virus inputs (Figure 6B). No inhibition of infection and replication was observed for YU-2 (Figure 6C), which expresses a gp120 that does not bind α4β7 [35].

**Table 2:** Description of T/F and chronic infectious molecular clones (IMCs).

IMC type	Subject	IMC name	Country	Risk factor^a^	Sex	Viral load (RNA copies/ml)	Fiebig stage^b^	Env AA 179–181^c^	V1V2 PNGs^d^	Reference
T/F	704010042^e^	CH042	South Africa	HSX	M	181,000	IV	LDI	5	in prep^f^
	705010067	CH067	South Africa	HSX	F	639,000	I/II	PDI	5	in prep^f^
	705010162	CH162	South Africa	HSX	M	13,100,000	III	LDI	6	in prep^f^
	703010131	CH131s	Malawi	HSX/MSM	M	411,873	I/II	LDL	5	in prep^f^
	ZM246F^e^	ZM246F-10^e^	Zambia	HSX	F	10,013,800	II	LDI	3	21
	ZM249M^e^	ZM249M-1^e^	Zambia	HSX	M	>2,000,000	IV	LDI	6	21
Chronic	703010256	CH256	Malawi	HSX	F	28,066	n/a	LDV	7	this study
	702010432^e^	CH432^e^	Malawi	HSX	M	40,570	n/a	LDI	8	this study
	707010457^e^	CH457^e^	Tanzania	HSX	F	234,671	n/a	VDI	6	this study
	705010534^e^	CH534^e^	South Africa	HSX	F	63,300	n/a	LDI	5	this study

a HSX = heterosexual exposure; MSM = Men who have sex with men.

b defined in [40]; n/a = not applicable for subjects enrolled in chronic cohorts.

c amino acid sequence of α4β7-binding tripeptide in V2 (positions 179–181 in HXB2).

d potential N-linked glycosylation sites in the V1V2 region (also see Figure S5).

e Env from subject also tested, see Table 1.

f Kappes et al., manuscript in preparation.

**Figure 7:**
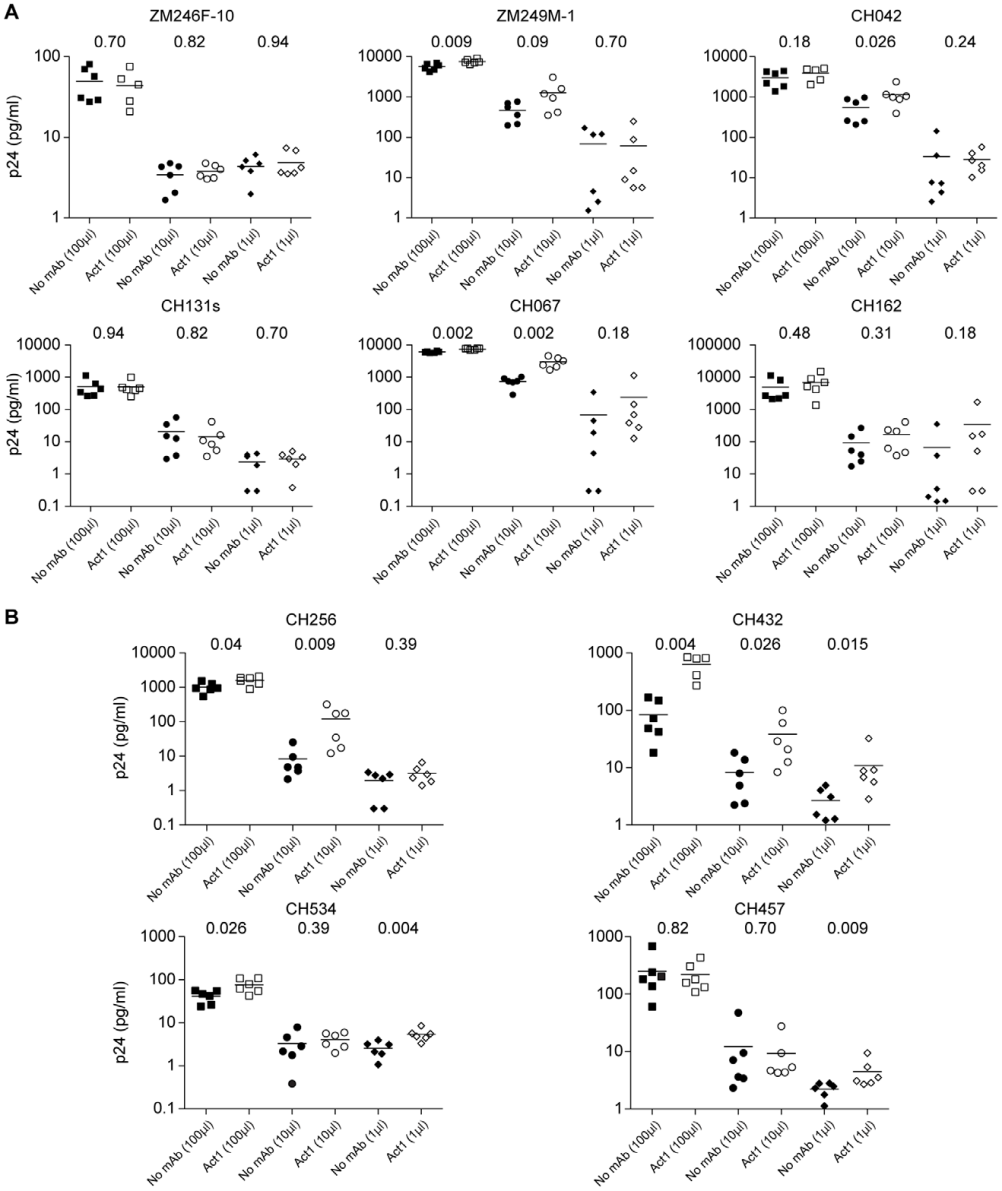
Blocking α4β7 does not inhibit replication of subtype C T/F and chronic IMCs. CD4+ T cells were infected at three different multiplicities using CD4+ T cell derived virus stock (1 µl, 10 µl and 100 µl). Virus replication was monitored by measuring p24 content in culture supernatants; each p24 measurement was repeated two to four times. The average p24 value in each of six independent wells at six days post-infection is plotted on the *y*-axis, with the bar representing the mean p24 value. Uncorrected Mann-Whitney p-values comparing no mAb to Act1 pre-treatment are shown for each viral input. Blocking α4β7 with Act1 increased p24 production for some viruses, but had no reproducible inhibitory effect on any T/F (**A**) or chronic (**B**) molecular clones tested, under conditions where reproducible inhibition of NL4-3-SF162 was achieved, as previously described [34].

If mucosal transmission selects for viruses that interact with α4β7, we reasoned that the replication of T/F IMCs would be inhibited when the Env-α4β7 interaction was blocked. Our subtype C IMC infection assays were powered to detect a 30% or higher decrease in virus (p24 antigen) production on day six, a time point when the largest effect on virus growth following α4β7 blockade was observed in previous experiments [34]. Two to four ELISA measurements were performed to monitor virus production in each of six replicate wells infected at different multiplicities using CD4+ T cells from three different donors. At the lowest virus inoculum, replication was undetectable in one to four of the six replicate wells from each of the ten subtype C viruses, indicating that virus was added at limiting dilution. Using Act1 at saturating concentrations, we observed no significant inhibition of replication of any subtype C virus (T/F or chronic) at any viral inoculum or time point post-infection (Figure 7), while replication of the positive control SF162 was reduced. In addition to Act1, we tested the α4 integrin-specific mAb 2B4 using SF162 and three of the subtype C viruses in CD4+ T cells from two donors and obtained similar results: a modest and transient inhibition of SF162, but no inhibition of the other viruses (data not shown). Finally, we tested seven subtype B T/F IMCs [37] using cells from a single donor and again observed no inhibition using the anti-α4β7 mAb Act1 (data not shown). Taken together, these results indicate that blocking the integrin α4β7 does not reduce the replication of T/F and chronic subtype C viruses in atRA-stimulated primary CD4+ T cells.

While Act1 failed to inhibit infection and/or replication by any of 13 T/F and four chronic control viruses, it significantly increased p24 production of five of the ten subtype C viruses (two of six T/F and three four chronic viruses; Figure 7). This was observed at multiple time points and with multiple viral inputs. To determine whether antibody binding to α4β7 could lead to enhanced cellular activation [69] and a resulting increase in virus production, we examined the expression of cellular activation markers. We found that neither Act1 nor 2B4 increased the expression of CD25, HLA-DR, Ki67, and CD69 at 1 hour, 2 days, and 5 days post-treatment, nor did these antibodies lead to an increase in CCR5 or α4β7 expression levels (Figure S6). However, we noted increased clumping of cells in both Act1 and 2B4 treated cultures [70], which raised the possibility that the enhanced virus production seen in some Act1-treated cultures could be due to increased cell-to-cell viral spread. We thus used high-speed cell imaging to examine cell-cell conjugates in CD4+ T cell cultures from a single donor that were infected with SF162 as well as three subtype C viruses (2 T/F and 1 chronic control) after treatment with Act1, 2B4 or a murine IgG1 isotype-control. Neither Act1 nor 2B4 increased expression of the high-affinity form of LFA-1, which is known to be upregulated by α4β7 engagement of gp120 [33]. However, we noted a significant increase in the expression of the cell-cell adhesion molecule ICAM-1 in cells exposed to 2B4 or Act1 compared to murine IgG1 (Figure S7A). Consistent with increased cell-cell spread, more cell-cell conjugates were virus-positive than predicted, with doublets being more than twice as frequently infected as singlets (p = 0.04) and triplets being more than three times as frequently infected as singlets (p = 0.06) (Figure S7B). Overall, these results suggest that Act1- and 2B4-mediated increases in cell-cell conjugates could facilitate more efficient spread and replication of some viruses in the absence of increased cellular activation.

## Discussion

The identification of viral traits that might enhance mucosal transmission is an important goal for vaccine development and other prevention strategies. A first step in characterizing such traits is the identification of T/F viruses, while a second step is the selection of appropriate chronic controls. Virological traits that are strongly associated with transmission, such as CCR5 use, should be readily identifiable when comparing T/F viruses to virtually any control group, while identifying more subtle phenotypes will greatly depend on the choice of control viruses, perhaps explaining discrepancies in genetic and phenotypic transmission signatures identified by different groups [17], [18], [21], [23], [27]–[30], [32]. Finally, the use of *in vitro* assays that recapitulate key steps in mucosal transmission are needed to identify properties unique to T/F viruses. Here, we have compiled a relatively large panel of both Envs and IMCs representing subtype C T/F and chronic control viruses, and developed a series of infection assays using virus pseudotypes, replication competent viruses, cell lines and primary human CD4+ T cells to improve our ability to identify viral phenotypes associated with transmission.

Env glycoproteins of HIV-1 can differ significantly in the efficiencies with which they utilize CD4 and the viral coreceptors, which in turn can impact viral tropism [45]–[49]. Given the variability in expression levels of entry cofactors on different cell subsets as well as between individuals [71]–[73], it is easy to envision several ways in which Env function could impact transmission efficiency at the level of virus entry. To date, several genetic Env signatures have been reported, with more compact variable loop structures and fewer PNGs being the most frequent findings [27]–[30], [32]. It is possible that these genetic traits impact Env function in ways that increase transmission fitness. However, to date no consistent T/F phenotype has been described. Thus, it is possible that mucosal transmission is a stochastic event where any reasonably functional R5 or dual tropic Env can initiate a productive infection [74]. However, it is also possible that the *in vitro* assays employed thus far have failed to reveal subtler or more transmission-specific phenotypic differences. The recent finding that some gp120 proteins from early HIV-1 infections can bind to the α4β7 integrin is consistent with this, although the ability of T/F viruses to productively interact with α4β7 was not explored.

To determine whether subtype C T/F viruses, which account for the great majority of new infections worldwide, utilize CD4 or CCR5 with enhanced efficiency, we tested both T/F and chronic Env constructs in pseudotyping assays. Consistent with previous results for subtype B [20] and subtype C [18], we failed to observe differences in both CCR5 and CD4 utilization. This is in contrast to findings by Etemad and colleagues who reported enhanced CCR5 utilization by Envs from individuals with chronic subtype A infection, although only V1–V5 fragments were tested in the context of chimeric viruses [23]. Similarly, Nawaz and colleagues found that gp120s from three subjects acutely infected with subtype A and C viruses bound to dodecameric but not monomeric CD4, while gp120s from subsequent time points of two of the same subjects bound to CD4 in both forms, suggesting an increase in CD4 affinity in later stages of infection [35]. These results may be specific to the particular Envs [35] or Env fragments [23] used, or due to the fact that gp120 and particle-associated Env trimers bind CD4 differently. In either case, current data utilizing a large number of Env constructs strongly suggests that the mucosal bottleneck is not the result of selective transmission of viruses with highly efficient CD4 or CCR5 use [17], [18], [20], or with increased efficiency of entry into particular CD4+ T cells subsets [20].

We also examined whether subtype C T/F and chronic Envs differed in their interaction with the gut homing integrin α4β7 as recently proposed [35], although previous data are almost entirely based on gp120 binding studies. In many ways, the α4β7 hypothesis is an attractive one. This integrin is expressed at high levels on activated CD4+ T cells in the gut [62] and cervicovaginal mucosa [63], both representing major sites of HIV replication early in infection [75], [76]. Moreover, intravenous administration of an anti-α4β7 mAb in rhesus macaques prior to and during acute infection with SIVmac239 resulted in decreased virus loads, perhaps by inhibiting trafficking of α4β7-positive T cells to the GI tract [77]. Finally, gp120-induced α4β7 signaling could promote virus replication through increased cell-to-cell adhesion. However, the ability of HIV-1 gp120 to bind α4β7 is far from universal - the commonly studied subtype B gp120s examined to date either do not bind to α4β7 or do so weakly, with the exception of SF162 [35]. Nonetheless, several gp120 proteins derived from early subtype A or subtype C infections have been shown to exhibit α4β7 binding capacity, and there is an obvious link between some α4β7 binding properties (fewer PNGs in the V1–V4 region) and genotypes associated with virus transmission in subtype C viruses [21], [27], [35]. While monomeric gp120 binds CD4, viral coreceptors and most broadly neutralizing antibodies, it differs from virion-associated Env trimers in important ways. Perhaps the best example is that numerous antibodies that bind to gp120 fail to neutralize the cognate Env trimer, consistent with both conformational differences and the fact that certain gp120 domains are sequestered in the oligomeric molecule. Thus, a key question that remains relatively unexplored is whether α4β7 binding by gp120 translates into an interaction by trimeric Env that influences virus infection and spread. To address this question, we concentrated on virus infection assays rather than gp120 binding experiments.

In our attempts to define the role of α4β7 in HIV-1 transmission, we were able to replicate a previous key finding, namely that saturating levels of antibodies to α4β7 modestly suppressed infection and replication by the prototypic subtype B strain HIV-1/SF162 [33], [34]. The inhibitory effects of α4β7 antibodies on SF162 infection were both transitory and most evident when low levels of virus input were used, which is precisely what would be expected if α4β7 functioned as an attachment factor [34], [35]. Attachment of virus particles to the host cell surface is a significant rate-limiting step to virus infection *in vitro*, but can be overcome in part by spinoculation [65], the inclusion of polycations that enhance viral binding [78], or the expression of virus attachment factors such as CD209 or CD209R [79], [80]. In the case of attachment factors, their ability to enhance infection is most pronounced when low levels of virus are used. Thus, our finding of a partial inhibition of SF162 replication in α4β7-positive T cells six days post infection at the lowest virus input is entirely consistent with previous reports and shows that our assays are sufficiently sensitive to measure the impact of α4β7 blockade on virus infection. Despite this, we found no inhibition of any T/F or chronic subtype B or C virus using cells from multiple donors and levels of virus empirically determined to be barely sufficient to establish a spreading infection. These findings are consistent with those of Pauls and colleagues, who found that a mAb to α4 used for the treatment of multiple sclerosis and Crohn's disease did not impact infection of atRA-treated CD4+ T cells by several HIV-1 strains, including two with the LDI/V tripeptide binding motif in the V2 region [64]. Since most of our T/F and chronic viruses possessed the α4β7-binding tripeptide motif as well as below average numbers of PNGs in the V1/V2 region, selection bias - i.e. the preferential inclusion of viruses that would be unlikely to interact with α4β7 - can also be excluded. Thus, we favor the hypothesis that not all Envs that can bind α4β7 in the form of gp120 necessarily do so as unliganded trimers.

Our failure to detect enhancement of viral infection of human CD4+ T cells by primary subtype B or C viruses, including T/F viruses, due to α4β7 interaction is by no means definitive, but does suggest that extrapolating results from gp120 binding assays to more complex virion infectivity studies may be misleading. It is possible that α4β7 interactions will be more important in other types of infection assays. In addition, we have not tested the ability of gp120 proteins derived from our viruses to bind to α4β7, although the relevance of such findings remains uncertain unless the corresponding trimeric Env exhibits similar properties. Our results demonstrate the importance of using replication-competent viruses to study properties associated with mucosal transmission. In contrast to single-round pseudovirus assays, experiments with IMCs are unbiased with respect to the genes that could influence fitness and enable detection of subtle differences following multiple rounds of replication. Thus, T/F and chronic IMCs are ideal reagents for future studies of phenotypes that may influence HIV-1 transmission.

## Materials and Methods

### Study subjects

This study was conducted according to the principles expressed in the Declaration of Helsinki. It was approved by the Institutional Review Boards of the University of Pennsylvania and Duke University. All subjects provided written informed consent for the collection of samples and subsequent analysis. Blood samples were obtained from 26 subjects infected with HIV-1 subtype C. A summary of their geographic origin and infection status is shown in Table 1. Blood specimens were collected in acid citrate dextrose, and plasma was separated and stored at −70°C.

### Generation of T/F and chronic Envs and IMCs

The inference and cloning of T/F Envs and IMCs from SGA-derived viral sequences has been described (Figure S1) [10], [12], [13], [25], [37]. To ensure efficient expression of cloned subtype C Envs for pseudotyping, the sense primer used for amplification of the corresponding *rev1-vpu-env* cassette lacked the *rev* initiation codon (underlined) (5′-*CACC*GGCTTAGGCATCTCCTATAGCAGGAAGAA-3′) [39].

Since chronic HIV-1 infections represent complex quasispecies of genetic variants, it is impossible to predict, based on sequence analysis alone, which members of this quasispecies are functional and which are defective or partially defective. To generate biologically relevant chronic controls, we thus targeted viral variants for both Env and IMC construction that exhibited evidence of a recent clonal expansion. Viral RNA was extracted from the plasma of chronically infected individuals and subjected to SGA and direct amplicon sequencing as described [12], [13], with the following modifications: 5′ half genome amplification: 1^st^ round sense primer 2010ForRC 5′- GTCTCTCTAGGTRGACCAGAT -3′, 1^st^ round antisense primer 2010Rev1C 5′- AAGCAGTTTTAGGYTGRCTTCCTGGATG -3′, 2^nd^ round sense primer 2010R1C 5′- TAGGTRGACCAGATYWGAGCC -3′ and 2^nd^ round antisense primer 2010Rev2C 5′- CTTCTTCCTGCCATAGGAAAT -3′; 3′ half genome: 1^st^ round sense primer 07For7 5′- CAAATTAYAAAAATTCAAAATTTTCGGGTTTATTACAG -3′, 1^st^ round antisense primer 2.R3.B6R 5′- TGAAGCACTCAAGGCAAGCTTTATTGAGGC-3′, 2^nd^ round sense primer VIF1 5′- GGGTTTATTACAGGGACAGCAGAG -3′ and 2^nd^ round antisense primer Low2C 5′- TGAGGCTTAAGCAGTGGGTTCC -3′. Thermal cycling conditions were identical to [13] except that 60°C was used for primer annealing. Sequences were then aligned using ClustalW [81] and subjected to phylogenetic analysis using PhyML [82]. Phylogenetic trees were inspected for clusters of closely related viruses, or “rakes”, which are indicative of a recent clonal expansion. (Figures 1, S2 and S3). In five subjects (Table 1), at least one *env* amplicon was identical in sequence to the inferred “rake” consensus and thus selected for cloning using the pcDNA3.1 Directional Topo Expression kit (Invitrogen). In two subjects, observable “rakes” were limited to only two closely related sequences, which encoded Env proteins that differed by a single amino acid. In these cases, the amplicon that matched the within patient consensus at this ambiguous site was cloned. In the remaining six subjects, the consensus sequences of the clonal expansion “rakes” were chemically synthesized and cloned (designated .synR1 in Table 1). IMCs from chronically infected subjects (CH256, CH432, CH457, and CH534) were generated using the same approach. 3′ and 5′ half-genome SGA was performed using viral RNA from subjects with evidence of clonal expansion as determined by *env* sequencing. 3′ and 5′ half genome sequences were used to construct neighbor joining trees (Figure S3), and clusters of closely related sequences were selected for further analysis. A consensus sequence of the members of such “rakes” was generated using Consensus Maker (hiv.lanl.gov). 3′ and 5′ half genome sequences were confirmed to be identical in their 1,040 bp overlapping regions, chemically synthesized in fragments bordered by unique restriction enzymes, and ligated together to construct infectious proviral clones.

### Pseudovirus production

Virus pseudotypes were produced by co-transfecting 6 µg of pcDNA3.1^+^ containing the desired *env* clone with 10 µg of HIV-1 backbone (pNL43-ΔEnv-vpr^+^-luc^+^ or pNL43-ΔEnv-vpr^+^-eGFP (catalog no. 11100 from the NIH Aids Research and Reference Reagent program (ARRRP) contributed by Haili Zhang, Yan Zhou, and Robert Siliciano [83]) into 293T cells using the calcium phosphate precipitation method. 72 h post-transfection, the pseudovirus-containing supernatant was filtered through a 22 µm filter, aliquoted, and stored at −80°C. Pseudovirus used to infect primary CD4+ T cells was concentrated by ultracentrifugation through a 20% sucrose cushion. Pelleted pseudovirus was then resuspended in phosphate-buffered saline (PBS) in 1/100^th^ the initial volume. All luciferase-encoding pseudoviral stocks were serially two-fold diluted and used to infect NP2 cells to define the linear range of the assay.

### Coreceptor tropism testing and cell line infections

NP2 cells stably expressing CD4 and either CCR5 (NP2/CD4/CCR5) or CXCR4 (NP2/CD4/CXCR4) were infected with luciferase-encoding HIV-1 pseudoviruses by spinoculation in 96-well plates at 450× g for 90 min at 25°C. Cells were lysed with Brite-Glo (Promega) at 2 h post-infection and analyzed on a Luminoskan Ascent luminometer. To assess sensitivity to coreceptor inhibitor maraviroc, NP2/CD4/CCR5 cells were preincubated for 30 min with saturating concentrations of the CCR5 inhibitor maraviroc (1 µM; ARRRP catalog no. 11580; [84]) or the fusion inhibitor enfuvirtide (10 µg/ml) prior to infection. To assess sensitivity to broadly neutralizing mAbs (HIV-1 gp120 mAb IgG1 b12 (ARRRP catalog no. 2640) from Dennis Burton and Carlos Barbas [85]; HIV-1 gp120 mAb VRC01 (ARRRP catalog no. 12033) from Dr. John Mascola [60]; HIV-1 mAbs PG9 and PG16 (ARRP catalog no. 12149 and 12150) from IAVI [86]), viral pseudotypes were preincubated with 10 µg/ml of antibody for 1 hour at 37°C. Virus and antibody mixes were then used to infect NP2/CD4/CCR5 cells. All NP2 cell line infections were done in at least triplicate in at least three independent experiments using R5-tropic JR-FL as a positive control and Env-deficient pseudotypes as a negative control.

### CD4 utilization efficiency

The ability of Env pseudoviruses to infect cells expressing low levels of CD4 was determined using affinofile cells, which are a modified 293T cell line that stably express CD4 and CCR5 under the control of independently inducible promoters [50]. 5×10^2^ cells were plated in each well of a 96-well plate and allowed to grow for two days prior to infection. Cells were induced with 2 µM ponasterone, which induces supraphysiologic levels of CCR5 thus ensuring CD4 is the limiting factor in entry, and either 0.156 ng/ml (CD4-low) or 5 ng/ml (CD4-high) minocycline 18 hours prior to infection. Expression levels were monitored by quantitative FACS analysis [57]. At the time of infection, media was exchanged and 25 µl of luciferase-encoding pseudovirus was added. Cells were then spinoculated at 450 g for 90 minutes. Luciferase activity was measured three days post-infection. Each infection condition was done in at least triplicate in each of three independent experiments. Pseudovirus-containing vesicular stomatitis virus glycoprotein (VSV-G) which is CD4-independent and thus infects CD4-high and CD4-low cells equally, HIV-1 JR-CSF Env which requires high levels of CD4, and HIV-1 JR-FL Env which can utilize low levels of CD4 were included in all experiments as controls [87]. To calculate CD4-use efficiency, mean relative light units in CD4-low cells were divided by the value obtained in CD4-high cells. The signal-to-noise ratio was higher in affinofile cells than NP2/CD4/CCR5 cells. Nine Envs (5 T/F, 4 chronic) infected maximally-CD4-induced afffinofile cells less than 100-fold above background. For these Envs, we noted increased variability across independent assays. Additionally, the ratio of CD4-high to CD4-low infection was likely falsely elevated due to infection of CD4-low cells at background levels. These Envs are highlighted in Figure 2 and were excluded from subsequent analyses.

### Maraviroc IC_50_

Maraviroc IC_50_ values were determined by pretreating NP2/CD4/CCR5 cells with 11 serial 3-fold dilutions of maraviroc, ranging from 5.9 µM to 0.1 nM, or no drug then spinoculating as above with luciferase-encoding pseudovirus and measuring luminescence 72 h post-infection. NP2 cells were chosen for this experiment because in the absence of CCR5 (NP2/CD4 only) these cells are highly restrictive to infection [88]; thus entry through potential alternative coreceptors when CCR5 is blocked by maraviroc is negligible. IC_50_ was determined using Prism 4.0 to determine the best fitting non-linear curve. Reported IC_50_ values are the mean of four independent experiments, with each drug concentration/pseudovirus condition performed in duplicate.

### Primary human CD4+ T cell tropism assay

Primary human CD4+ T cells were purified by negative selection by the University of Pennsylvania's Human Immunology Core. Cells were stimulated with plate-bound anti-CD3 (clone OKT3) (eBiosciences) and anti-CD28 (clone 28.2) (BD Biosciences) and 20 U/ml recombinant interleukin-2 (IL-2) (aldesleukin, Prometheus Laboratories) in RPMI supplemented with 10% fetal bovine serum (FBS, Sigma-Aldrich). Three days after stimulation, cells were transferred to 96-well V-bottom plates. Five microliters of concentrated GFP-expressing pseudovirus was used to infect 6.7×10^5^ cells in triplicate by spinoculating at 1,200× *g* for 2 hours. Cells were then transferred to new 24-well plates, and new medium containing 20 U/ml IL-2 was added. Three days post-infection, cells were stained for flow cytometry [20], [54].

### Flow cytometry

A total of 1–2×10^6^ cells from each condition were stained for flow cytometry. Incubations were done at room temperature in fluorescence-activated cell sorter (FACS) wash buffer (PBS, 2.5% FBS, 2 mM EDTA). Cells were first washed in PBS, then live/dead Aqua (Invitrogen) was added and incubated for 10 min. Next, anti-CCR7 IgM (BD) in FACS wash buffer was added and incubated for 30 min. Cells were then washed in FACS wash buffer before staining with anti-CD3–Qdot 655 (Invitrogen), anti-CD4–Alexa Fluor 700 (BD), anti-CD45RO–phycoerythrin (PE)-Texas Red (Beckman Coulter), and anti-IgM–PE (Invitrogen) for 30 min. Cells were then washed in FACS wash buffer and resuspended in 1% paraformaldehyde (PFA). Samples were run on an LSRII (BD) instrument and analyzed with FlowJo 8.8.6 (Treestar). Cells were gated as follows: singlets (FSC-A by FSC-H), then live cells (SSC-A by live/dead), then lymphocytes (SSC-A by FSC-A), then CD3^+^ cells (SSC-A by CD3), then memory markers (CCR7 by CD45RO).

To examine activation of cells treated with Act1, 2B4, isotype control murine IgG1, or no antibody, cells were pretreated with 33 nm of the specified antibody for 1 hour, 2 days, or 5 days. At each time point, 1×10^6^ cells were washed in PBS, then live/dead Aqua was added for 10 minutes. Anti-CCR5-PE (BD), anti-CD4-PerCP-Cy5.5, anti-CD25-APC-Cy7, anti-HLA-DR-PE-Cy7, anti-α4β7-Alexa Fluor 680 (clone Act1) and anti-CD45RO-TexasRed-PE were added in FACS wash buffer for 30 minutes. Cells were then washed in FACS wash buffer and treated with cytofix/cytoperm buffer (BD) for 17 minutes. Anti-CD3-V450, anti-CD69-APC, and anti-Ki67-FITC in perm/wash buffer were added and incubated for 1 hour at room temperature. Cells were washed in perm/wash buffer, fixed in 0.1% paraformaldehyde, run on an LSRII instrument and data was analyzed with FlowJo. Live cells expressing CD3 and CD4 were analyzed for expression of activation markers.

### Production of HIV in primary CD4+ T cells

Replication competent viral stocks were generated by transfecting a 10 cm dish 30% confluent with 293T cells with 6 µg of IMC DNA. Virus was harvested 72 hours post-transfection and filtered through a 45 micron low protein binding filter. 293T-derived HIV was then used to infect stimulated human CD4+ T cells. 18 hours after infection cells were washed twice to remove unbound 293T-derived virus. CD4+ T cell derived HIV was then harvested 11 days post infection, filtered through a 45 micron filter, aliquoted and frozen at −80°C. p24 antigen concentration of viral stocks was assessed by Alliance ELISA and high sensitivity alphaLISA (Perkin Elmer); these methods were in good agreement for all IMCs tested.

### Infection assay to assess effect of α4β7 blockade

Freshly isolated human CD4+ T cells purified by negative selection were stimulated with 1.5 µg/ml anti-CD3 clone OKT3, 20 units/ml IL-2, and 10 nM atRA. atRA was resuspended in DMSO, filter sterilized, aliquoted in the dark, and immediately frozen at −80°C for no longer than one month. 24 hours after stimulation, media was removed, and new media with IL-2 and atRA was added. Media was changed and new IL-2 and atRA were added every two to three days. Efforts were made to precisely follow previously reported methods [33], [34]. Cells were infected six days post-stimulation for both pseudotype and replication competent HIV infection. Cells were pre-treated for 1 hour with 33 nM Act1 (an α4β7 heterodimer specific mAb; ARRRP catalog no. 11718 from Dr. A. A. Ansari [89]) at 37°C prior to infection. α4β7 expression and saturating mAb blockade was confirmed on the day of infection by flow cytometry. α4β7 expression was determined with Alexa Fluor 680-conjugated Act1 (Invitrogen). GFP-expressing pseudotype infections were performed as described above. SF162 and JR-FL pseudovirions were used as positive and negative controls, respectively [35]. For infections with replication competent HIV, virus made in CD4+ T cells was used to limit potentially non-physiologic properties of 293T-derived HIV. 2×10^5^ stimulated atRA-treated CD4+ T cells were plated in 100 µl per well of a 96-well plate. After incubation with Act1 or media only, 100 µl of CD4+ T cell-derived virus was added at a neat, 1∶10, or 1∶100 dilution. Cells were infected for five hours at 37°C without spinoculation and then cells were washed four times to remove unbound HIV. Three, six, and nine days post-infection, media was changed and p24 antigen concentration was assessed in the supernatant using an alphaLISA high sensitivity kit (Perkin Elmer) read on a Synergy H4 plate reader (BioTek instruments). Either four or six replicate wells were used per condition, and each alphaLISA measurement was performed 2–4 times. AlphaLISA assays were performed in 25 µl volume in 384 well plates. Each plate contained an internal standard curve ranging four orders of magnitude with each standard concentration repeated in eight wells. The lower limit of detection for most assays ranged between 3 and 10 pg p24 per mL.

### High-speed cell imaging

Purified CD4+ T cells were treated with atRA, anti-CD3, and IL-2, and infected as described for α4β7 blocking experiments. Nine days post infection, cells exposed to 10 µl of NL4-3-SF162, ZM249, CH162, CH256, and mock infected were pooled from six replicate wells, washed in PBS and then FACS wash buffer. Anti-CCR5-PE-Cy7, anti-CD54 (ICAM-1)-PE-Cy5, anti-LFA-1-PE (clone MEM-148), and anti-α4β7-Alexa Fluor 680 (clone Act1) were added in FACS wash buffer and allowed to incubate for 30 minutes. Cells were then washed in FACS wash buffer and treated with cytofix/cytoperm buffer for 17 minutes. Anti-Gag-FITC (clone KC-57) was added for 1 hour. Cells were fixed in 1% paraformaldehyde and DAPI was added 30 minutes prior to analysis. Samples were run on an ImageStream IS100 equipped with two cameras and 405, 488, and 658 nm excitation lasers. At least 20,000 images were collected per condition, and the upper limit of images classified as cells was set to 600 pixels to allow collection of cell-cell conjugates. Cells were gated as follows: singlets, doublets, and triplets (DAPI by brightfield area), then Gag+ (Gag intensity by Gag median pixel). Gag positivity was gated such that the percent of mock-infected Gag+ cells was equal in singlet, doublet, and triplet populations. A single Gag-expressing cell was sufficient for a doublet or triplet image to be considered Gag+, as confirmed by visual inspection of Gag+ images. LFA-1 and ICAM-1 expression were analyzed on nucleated (DAPI+), focused (brightfield gradient root mean square-high), singlets (brightfield aspect ratio ∼1) to ensure that all analyzed images were of high quality.

### Statistical analysis

To test the hypothesis that T/F Envs as a group were different from chronic Envs in various functional measures, we used two-tailed Mann-Whitney tests. To test the hypothesis that Act1 treatment inhibited viral replication in CD4+ T cells, we again used two-tailed Mann-Whitney tests comparing the six biological replicates with and without Act1 treatment for each virus. No attempts were made to correct for multiple testing, largely because Act1 treatment did not have the expected effect on T/F viruses. Because six values from each group were compared, the minimum uncorrected *p*-value was 0.002. Applying the conservative Bonferroni correction would render all comparisons insignificant (α level of 0.05 divided by 39 tests = 0.001). Thus, we conclude that Act1 inhibits or enhances only when multiple input levels of the same virus show a consistent effect. To determine if cell-cell conjugates were infected more than expected by chance, the percent of Gag+ doublets was compared in a paired *t*-test to double the percent Gag+ singlets, and the percent of Gag+ triplets was compared to triple the percent Gag+ singlets.

### Nucleotide accession numbers

All newly obtained HIV-1 sequences have been submitted to GenBank and are available under accession numbers listed in Table S1.

## Supporting Information

Figure S1 **Inference of T/F** ***env*** **sequences.** Maximum likelihood trees of SGA-derived *env* sequences (left) and corresponding Highlighter plots (right) are shown for eight subjects acutely infected with HIV-1 subtype C viruses. Highlighter plots depict sequence alignments (Highlighter v2.1.1; hiv.lanl.gov); tick marks indicate differences compared to the top sequence (red, T; green, A; blue, C; orange, G; grey, gap). The trees were constructed using PhyML [82]. Except for in panel A (bar = 0.005 substitutions per site), the scale bar represents a single nucleotide substitution. Sequences chosen for cloning are marked with an arrow. In most subjects, a single low diversity lineage was observed (**B, D, E, F, H**), indicating infection with a single T/F virus. Subjects 20258279 (**A**) and 21197826 (**C**) were infected with four and two T/F viruses, respectively. The tree from subject 19157834 (**G**) shows a lineage with at least four shared mutations, which likely represents a second closely related T/F virus (a single sequence highlighted in green exhibits G→A hypermutation). In all cases, the cloned *envs* represent unambiguously determined T/F viruses. GenBank accession numbers for the *env* sequences are listed in Table S1.
(EPS)

Figure S2 **Identification of clonally expanded viral lineages in chronically infected individuals.** The phylogenetic relationships of SGA-derived *env* sequences depicting the quasispecies complexity in chronically infected subjects are shown (**A–J**). Trees were constructed using maximum likelihood methods [82]. Asterisks indicate bootstrap support of greater than 80% (the scale bar represents 0.02 substitutions per site). Sequences highlighted in blue indicate a recently expanded viral lineage, the consensus sequence of which approximates their most recent common ancestor. Red arrows denote *env* amplicons chosen for cloning (**A, B, D, F, G, H, J**) while red lines indicate “rakes” used to infer consensus sequences for chemical synthesis (**C, E, I**). GenBank accession numbers for the *env* sequences are listed in Table S1.
(EPS)

Figure S3 **Construction of chronic IMCs from overlapping 5′ and 3′ half genome sequences.** The phylogenetic relationships of SGA-derived 5′ and 3′ half genome sequences depicting the quasispecies complexity in chronically infected subjects are shown. Trees composed of 5′ (**A, C, E, G**) and 3′ (**B, D, F, H**) half genome sequences were constructed using maximum likelihood methods [82]. Sequences highlighted in blue were used to generate half genome consensus sequences for each subject (**A** and **B** 703010256, **C** and **D** 702010432, **E** and **F** 707010457, **H** and **G** 705010534) that were confirmed to be identical in sequence in the region of overlap then chemically synthesized to generate full-length IMCs. Asterisks indicate bootstrap support of greater than 80% (the scale bar represents 0.02 substitutions per site). GenBank accession numbers for the 5′ and 3′ half genome sequences are listed in Table S1.
(EPS)

Figure S4 **Neutralization of T/F and chronic Envs.** The maximal percent inhibition (MPI) of 10 µg/ml of b12, VRC01, PG9 and PG16 on infection by T/F and chronic Env pseudoviruses is shown on the *y*-axis. The bar represents the median MPI. Numbers on the top indicate two-sided Mann-Whitney p-values comparing T/F to chronic Envs for each mAb.
(EPS)

Figure S5 **Alignment of V1V2 Env protein sequences.** An alignment of the first and second variable regions of the HIV-1 Env glycoprotein (HXB2 gp120 residues 131–196) is shown for previously described α4β7-reactive gp120s as well as T/F and chronic IMCs. Asparagine residues predicted to be glycosylated are shown in red. The α4β7 binding-site (consensus LDV/I; HxB2 residues 179–181) is highlighted in grey. Gaps, shown as dashes, were introduced to optimize the alignment.
(EPS)

Figure S6 **Act1 and 2B4 do not affect the activation profile of cells compared to a murine isotype control.** Cells from three different donors were treated with Act1 (red) or 2B4 (blue) as well as a murine IgG1 control (open) for one hour (**A**), two days (**B**), or five days (**C**), and analyzed for markers of cellular activation, including CD25, HLA-DR, Ki67, and CD69. The expression of CCR5 and α4β7 was also measured. The percent of live CD4+ T cells expressing each marker is shown on the *y*-axis. No significant differences were observed comparing Act1 or 2B4 treated cells to IgG1, except for decreased α4β7 expression at day five, which reflects decreased detection due to blocking with unlabeled Act1.
(EPS)

Figure S7 **Cell-to-cell adhesion and infection analysis.** High-speed imaging of single cells and cell aggregates was performed using an ImageStream IS100 cytometer. (**A**) Treatment with Act1 (red) or 2B4 (blue) increased the percent of singlet cells expressing the cell adhesion molecule ICAM-1 (y-axis) relative to a murine isotype (IgG, open) control (*p* = 0.04; Mann-Whitney). Each point represents cells from the same donor infected with a different virus, or mock infected; the bar indicates the group median. (**B**) Cells were gated based on DNA content and size into individual cells (singlets), two cells in contact (doublets) and three cells in contact (triplets). The percent of singlets, doublets, and triplets infected after exposure to different viruses is shown on the y-axis. Cell infection was determined based on Gag (p24) antigen expression compared to mock-infected control. Cell conjugates contained more infected cells than expected by chance, suggesting enhanced cell-to-cell spread (doublets, *p* = 0.04; triplets, *p* = 0.06; two-sided paired T-test).
(EPS)

Table S1 **GenBank accession numbers of sequences generated for this study.**
(DOC)
