# Supplementary material for: Transmitted/Founder and Chronic Subtype C HIV-1 Use CD4 and CCR5 Receptors with Equal Efficiency and Are Not Inhibited by Blocking the Integrin α4β7
Source: PLoS Pathog. 2012 May 31;8(5):e1002686. doi: 10.1371/journal.ppat.1002686 (PMC3364951; doi:10.1371/journal.ppat.1002686)
Supplement: Table S1 — GenBank accession numbers of sequences generated for this study. (DOC) [file ppat.1002686.s008.doc]

| **Infection stage** | **Subject** | ***env* clone accession numbers** | ***env* SGA accession numbers** | **5' half accession numbers** | **3' half accession numbers** |
| --- | --- | --- | --- | --- | --- |
| Acute | 20258279 | HQ595763 and HQ595764 (env) | JQ753730-JQ753770 | n/a | n/a |
|  | 2833264 | HQ595757 (env) | JQ754177-JQ754189 | n/a | n/a |
|  | 21197826 | HQ595753 (env) | JQ754190-JQ754202 | n/a | n/a |
|  | 21283649 | HQ595756 (env) | JQ754203-JQ754229 | n/a | n/a |
|  | 20927783 | HQ595750 (env) | JQ754230-JQ754240 | n/a | n/a |
|  | 1245045 | HQ595742 (env) | JQ754241-JQ754250 | n/a | n/a |
|  | 19157834 | HQ595743 (env) | JQ754251-JQ754286 | n/a | n/a |
|  | 2935054 | HQ595758 (env) | JQ754287-JQ754304 | n/a | n/a |
| Chronic | 704010330 | JQ777128 (env) | JQ777111-JQ777136 | n/a | n/a |
|  | 704010207 | JQ777073 (env) | JQ777061-JQ777086 | n/a | n/a |
|  | 702010141 | 702010141.synR1*^a^* (env) | JQ779286-JQ779324 | n/a | n/a |
|  | 703010180 | JQ777046 (env) | JQ777046-JQ777060 | n/a | n/a |
|  | 703010167 | 703010167.synR1*^a^* (env) | JQ779884-JQ779913 | n/a | n/a |
|  | 704010499 | JQ777164 (env) | JQ777144-JQ777164 | n/a | n/a |
|  | 704010461 | JQ777137 (env) | JQ777137-JQ777143 | n/a | n/a |
|  | 704010028 | JQ777039 (env) | JQ777026-JQ777045 | n/a | n/a |
|  | 703010269 | 703010269.synR1*^a^* (env) | JQ777165-JQ777194 | n/a | n/a |
|  | 704010273 | JQ777098 (env) | JQ777087-JQ777110 | n/a | n/a |
|  | 707010457 | 707010457.synR1*^a^* (*env*); CH457*^a^* (IMC) | n/a | JQ779148-JQ779169 | JQ779128-JQ779147 |
|  | 705010534 | 705010534.synR1*^a^* (*env*); CH534*^a^* (IMC) | n/a | JQ779206-JQ779225 | JQ779170-JQ779205 |
|  | 702010432 | 702010432.synR1*^a^* (*env*); CH432*^a^* (IMC) | n/a | JQ779256-JQ779285 | JQ779226-JQ779255 |
|  | 703010256 | CH256*^a^* (IMC) | n/a | JQ779098-JQ779127 | JQ779074-JQ779097 |

**Table S1.** GenBank accession numbers of sequences generated for this study

*^a^*chemically synthesized sequence reported below.

>702010141.synR1

ATGAGAGTGATGGGGATACAGAGGAATTGTCAACAATGGTGGATATGGGGAATCTTAGGCTTTTGGATGCTAATGATTTGTAATGGTAACTTGTGGGTCACAGTCTACTATGGGGTACCAATATGGAAAGATGCAAAAGCTACTCTATTTTGTGCATCAGATGCTAAAGCATATGAAAAAGAGGTACATAATGTCTGGGCTACACATGCCTGTGTCCCCACAGATCCCGATCCACAAGAAATGTTTTTGAAAAATGTAACAGAATATTTTAATATGTGGAGAAATGACATGGTGGATCAGATGCATGAAGACATAATCAGTTTATGGGATGAAAGCCTAAAACCATGTGTAAAGTTGACCCCACTCTGTGTTACTTTAAACTGTTCTATCATCTACCGAAATAAGACCAACCAAAATGACACGGAGAGTATCTACCCAAGTGACAAGGAAATACCTTACCAAAATGACACAGGGATAAGAAATTGCTCTTTCAATGCAACCACAGAAATAAGAGATAAGAAAAAGAAAGAAGGGGCACTTTTTTATAGACTTGATGTAGTGCCACTAGATGAAAATACCAACTCTACTCATAGGCCATATAGATTAATAAATTGTAATACCTCAGTTGTAACACAAGCCTGTCCAAAGGTCTCTTTTGACCCAATTCCTATACATTATTGTGCTCCAGCTGGTTATGCAATTCTAAAGTGTAATAATGAGACATTTGATGGAATGGGATTATGCAATAATGTCAGCACAGTACAATGTACACATGGAATTAAGCCAGTGGTATCAACTCAACTACTGTTAAATGGTAGCCTAGCAGAAAAAGAAATAATAATTAGCTCTGAAAATCTGACAAACAATGGCAAAAACATAATAGTACATCTCAATGAATCTGTAGAGATTGAGTGTATAAGACCCAACAATAATACAAGGAAAAGTGTGAGAATAGGACCAGGACAAACATTCTATGCAACAGGAGAGATAATAGGAGATATAAGAGAAGCATATTGTCACATTAATAAAACAAAATGGAATAGAACTTTAGAAATGGTAAGGGAAAAATTAAAGGAAAAATTCGAAAAACACTCCCCTAATAAAACAATAGAATTTAGAAATTCCTCAGGAGGGGACCTAGAAATTACAACACATAGCTTTAATTGTAGAGGAGAGTTTTTCTATTGCAATACATCAAGATTGTTTAGTAGTAATAGTACAGAAATTAATAGTACAGAAAGTTCCAATTCAACAATCATACTCCCATGCAGAATAAAACAAATTATAAATATGTGGCAGGGGGTGGGACGGGCAATATATGCAGCTCCTATTGCAGGAAAAATAACATGTAAATCAAATATCACAGGACTAATATTGACACGTGATGGAGGAGAGGGCAATAATACAGAAAACAGAAGTGAGATATTCAGACCTACAGGAGGAGATATGAGGGACAATTGGAGAAGTGAATTATACAAATACAAAGTAGTAGAAATTAAGCCATTAGGAGTAGCACCCACTAATGCAAAAAGGAGAGTGGTGGAGAGAGAAAAAAGAGCAGTGGGATTAGGAGCTATGTTCCTTGGGTTCTTGGGAATGGCCGGAAGCACTATGGGCGCAGCATCAATAACGCTGACGGTACAGGCCAGACAAGTATTGTCTGGTATAGTGCAACAGCAAAGCAATTTGCTGAGGGCTATAGAGGCGCAACAGCATATGTTGCAACTCACGGTCTGGGGCATTAAACAGCTCCAGGCAAGAGTCCTGGCCATAGAAAGATACCTAAAGGATCAACAGCTCCTAGGGATGTGGGGCTGCTCTGGAAAACGCATCTGCACCACTACTGTGCCTTGGAACTCTAGTTGGAATAATAGAAATTATAGTGATATTTGGGATAATCTGACATGGATGCAATGGGATAAAGAAATTAGTAATTACACAGACATAATATATGAGTTGCTTGAAAAATCACAAAACCAGCAGGAAGAAAATGAAAAAGATTTACTAGCATTGGACAGCTGGAACAATCTGTGGAATTGGTTTAGCATAACAAACTGGCTGTGGTATATAAAAATATTCATAATGATAGTAGGAGGCTTGATAGGTTTAAGAATAATTTTTACAGTACTCTCTTTAGTAAATAGAGTTAGGCAGGGATACTCACCTTTGTCGTTTCAGACCCTTACCCCAAACCGGAGGGAACTCGACAGGCTCGGAAGAATCGAAGAAGAAGGTGGAGAGCAAGACAGAAACAGATCCATTCGATTAGTGAGCGGATTCTTGTCACTTGCCTGGGAAGACCTGCGGAGCCTGTGCCTTTTCAGCTACCACCGATTGAGAGACTTACTATTGATTGCAGCGAGAGCAGTGGAACTTCTGGGACGCAGCAGTCTCAGGGGGCTGCAGAGAGGGTGGGAAGCTCTTAAGTATCTGGGAAGTCTTGTGCAATATTGGGGTCTGGAGCTAAAAAGGAGTGCTATTAGTCTGCTTGATACCATAGCAATAGCAGTAGGTGAAGGAACAGATAGGATTTTAGAATTAATACAAAGAATTTGTAGAGCTATCCGCAACATACCTAGAAGAATAAGACAGGGCTTAGAAGCAAGCTTGCTATAA

>703010167.synR1

ATGAAAGTGATGGGGAAATTGAGGAATTATCAACAATGGTGGATATGGAGCATCTTAGGCTTTTGGATGACTTATAGTGTGTTGGGGAACTTGTGGGTCACAGTCTATTATGGGGTACCGGTGTGGAGAGAAGCAAAAACTACTTTATTCTGTGCATCAGATGCTAAAGCATATGAGAAAGAAGTGCATAATGTTTGGGCTACACATGCCTGTGTACCCACAGACCCCAGCCCACAAGAAATGGTTTTAAAAAATGTAACAGAAAATTTTAACATGTGGAAAAATGATATGGTGGATCAGATGCATGAGGATATAATCAGTCTATGGGATCAAAGCCTAAAGCCATGTGTAAGGTTGACCCCACTCTGTGTCACCTTAAACTGTAGTAATGCTAATGTTAATGTTACTGATATCCACAATAATACCATGAAAGGAGAAGTAAAAAATTGCTCTTTCATGGCAACCACAGAAATAAAAGATAAGAAAAAACAAGTGTATGCACTTTTTTATAGACTTGATATAGTACAACTTAATGAGAGTAGTAATAACAGCTATGAGAGCTATAGATTAATAAATTGCAATACCTCAGCCATAACACAAGCCTGTCCAAAGGTCTCTTTTGATCCAATTCCTATACATTATTGTGCACCAGCTGGTTTTGCGATTCTGAAGTGTAATAATAAGACATTTAATGGAACAGGACCATGCAATAATGTCAGTACAGTACAATGTACACATGGAATTAAGCCAGTGGTATCAACTCAACTACTGTTAAATGGTAGTACAGCAGAAAAGGAGATAATAATCAGATCTGAAAATCTGACAAATAATGCCAAAACAATAATAGTACATCTTAATGAATCTGTAGAAATTGTGTGTATAAGGCCCGGCAATAATACAAGGAAGAGTATAAGAATAGGACCAGGACAAACATACTTTTCAACAGGAGAAATAATAGGAAATATAAGACAAGCACACTGTAACATTAGTGAAAGTAAATGGAATGAAACTTTACAAAAGGTAGGAAGAAAATTAGCAGAACACTTCCCTAATAAAACAATAATATTTAATTCTTCCTCGGGAGGGGACCTAGAAATTACAACACATAGCTTTAATTGTAGAGGAGAATTTTTCTATTGTAATACATCAGGACTGTTCAATAGTACATACAATAGTACATACAATGGTCCATACAATGGTACAGCAATAATCACACTCCCATGCAGGATAAAACAAATTATAAACATGTGGCAGGAGGTAGGCAGAGCAATGTACGCCCCTCCCATTGAAGGAAACATAACATGTAACTCAAGCATCACGGGACTACTCTTGGTACGTGATGGAGGCGAAACAAATAGTTCCCAAGAGGAGATATTCAGACCTGGAGGAGGAGATATGAGAGACAATTGGAGAAGTGAATTATATAAATATAAAGTGGTAGAAATTAAACCATTAGGAATAGCACCTACTGAGGCAAAAAGGAGAGTGGTGGAGAGAGAAAAAAGAGCAGTGGGAATAGGAGCTGTGTTCCTTGGGTTCTTGGGAGCAGCAGGAAGCACTATGGGCGCAGCATCAATAACGCTGACGGTACAGGCCAGACAATTGTTGTCTGGTATAGTGCAGCAGCAAAGTAATTTGCTGAGAGCCATAGAGGCGCAACAGCATATGTTGCAACTCACAGTCTGGGGCATTAAGCAACTCCAGACAAGAGTCCTGGCTATAGAAAGATACCTAAAGGATCAACAGCTCCTAGGGATGTGGGGATGCTCTGGAAAAATCATCTGCACCACCAACGTGCCTTGGAACTCCAGTTGGAGTAATAAATCTAAAAAAGACATTTGGGATAACATGACCTGGATGCAGTGGGATAAAGAAGTTAATAATTACACAGACATAATATACAGGTTGCTTGAAGAATCACAAAACCAGCAGGAAGAAAATGAAAAAGATTTACTAGCATTGGACAGTTGGAACAATCTGTGGAATTGGTTTAGCATAACAAAATGGCTGTGGTATATAAAAATATTCATAATGATAGTAGGAGGCTTGATAGGTTTAAGAATAATTTTTGCTGTGCTTTCTATAGTGAATAGAGTTAGGCAGGGATACTCACCTTTGTCGTTTCAGACCCTTATCCCAACCCCGAGGGAACCAGACAAGCTCGGAGGAATCGAAGAAGAAGGTGGAGGGCAAGACAGAGACAGGTCCGTGAGATTAGTGAGCGGATTCTTGTCACTATTCTGGGACGATCTGAGGAGCCTGTGCCTTTTCAGCTACCACCAATTGAGAGACTTTATATTGGTGACAGCGAGAGCAGCGGAACTTCTGGGACGCAGCAGTTTCAGGGGACTACAGAGGGGGTGGGAAGCCCTTAAATATCTGGGAAGTCTTGTGCAGTATTGGGGTCTAGAACTAAAAAAGGGGGCTATTAGTCTGCTTGATACCACAGCAATAACAGTAGCTGAAGGAACAGATAGAATTATAGAAGTACTGCAAAGACTTTGGAGAGCTATCTGCAACATACCTAGAAGAATAAGACAGGGCTTTGAAGCAGCTTTGCAATAA

>703010269.synR1

ATGAGAGTGATGGGGATACTGAGGAATTGTCAACGATGGTGGATATGGGGCATCTTAGGCTTTTGGATGGTAATGACTTATAGTGTGATGGGAAACTTGTGGGTCACAGTCTATTATGGGGTACCTGTGTGGAGAGAAGCAAAGGCTACTCTATTCTGTGCATCAGATGCTAAAGCACATGAAAGAGAAGTGCATAATGTTTGGGCTACACATGCCTGTGTACCCACAGACCCCAACCCACATGAAATGGTTTTGGAAAATGTAACAGAAAATTTTAACATGTGGAAAAATGACATGGTGGAGCAGATGCATAAGGATATAATCAGTTTATGGGACCAAAGCCTAAAGCCATGTGTAAAGTTGACCCCACTCTGTGTCACTTTGAACTGTGTCAATGCTAATGTTACCGGTAATGGTACTGCTAGCAGTAATGGTACTGCTAACAGTACTGGTACTGCTAACTATACTAATACCAGGATTGAAGAAATGAAAAATTGCTCTTTCAATACAACCACAGAAATACAAGATAAAAAAAGGAAAGAGCATGCACTTTTTTATAAACTTGATATAGTACCACTTAAAGAGAATGACAACAGCAGCTATAGGTTAATAAATTGCAATACCTCAGCCGTAACACAAGCCTGTCCAAAGGTCACTTTTGATCCAATTCCTATACATTATTGTGCTCCAGCTGGTTATGCGATTCTAAAGTGTAATAATAAGACATTCAATGGGACAGGACCATGCAATAATGTCAGCACAGTACAGTGTACACATGGAATTAAGCCAGTGATATCAACTCAATTAATATTAAATGGTAGCCTAGCAGAAAAAGAGATAATAATTAGATCTGAAAATCTGACAAACAATGTCAAAACAATAATAGTACATCTTAATGAATCTGTAGAGATTACGTGTATAAGACCCGGCAATAATACAAGAAGAAGTATGAGAATTGGACCAGGACAAACATTCTATGCAACAGGAGACATAATAGGAGATATAAGAAAAGCACATTGTAACATTAGTACAGCAAGTTGGAATAAAACTTTACAGAGGGTAAGAGAAAAATTAGAAGAACACTTCCCTAATAAAACAATAAGATTTGAGCCACCTGCAGGAGGGGACCTAGAAATTACAACACATAGCTTTAATTGTAGAGGAGAATTTTTCTATTGTAATACATCACGCCTGTTTAATAGTACATACAATGGTACAGACGGGTCTAATAGCACAACAAACATCACACTCCCATGCAGAATAAAACAAATTATAAACATGTGGCAGGAGGTAGGACGAGCAATGTATGCCCCTCCCATTGCAGGAAACATAACATGTAAATCAAATATCACAGGAATACTATTGACACGTGATGGAGGAGGGAACAGCACAGACCCAGAGATATTCAGACCTGGAGGAGGAGATATGAGGGACAATTGGAGAAGTGAATTATACAAATATAAAGTGGTAGAAATTAAGCCATTAGGAATAGCACCCACTACTGCAAAAAGGAGAGTGGTGGAGAGAGAGAAAAGAGCAGTGGGAATAGGAGCTGTGTTCCTTGGGTTCTTGGGAGCAGCAGGAAGCACTATGGGCGCGGCGTCAATAACGCTGACGGTACAGGCCAGACAATTATTGTCTGGTATAGTGCAACAGCAAAACAATTTGCTGAGAGCTATAGAGGCGCAACAGCATATGTTGCAACTCACAGTCTGGGGCATTAAGCAGCTCCAGGCAAGAGTCCTGGCTATAGAAAGATACCTAAAGGATCAACAGCTCCTAGGGATGTGGGGCTGCTCTGGAAAACTCATCTGCACCACTGCTGTACCTTGGAACAGTAGTTGGAGTAGTAAAAATCAAACGGCTATTTGGGATAACATGACCTGGATGCAATGGGATAGAGAAATTGAGAATTACACAAACATAATATACAATTTGCTTGAAGACTCGCAAATCCAGCAGGAAAAAAATGAAAAAGATTTACTAGCATTGGACAGTTGGAACAATCTGTGGAATTGGTTCAGCATAACAAAATGGCTAAGGTATATACAAATATTTATAATGATAGTAGGAGGATTAATAGGTTTAAGAATAATTTTTGCTGTGCTCTCTCTAGTAAATAGAGTTAGGCAGGGATACTCACCTTTGTCGTTTCAGACCCTTACCCCACACCCGAGGGGACCCGACAGGCTCGAAAGAATCGAAGAAGAAGGTGGAGAGCAAGACAAAGACAGATCCGTGCGATTAGTGAGCGGATTCTTAGCACTTGCCTGGGACGATCTGCGGAGCCTGTGCCTTTTCAGCTACCACCTATTGAGAGACTTTCTATTGATTGCAGCCAGAGTGGTGGAACAGGGACTGCAGAGGGGGTGGGAAGCCCTTAAGTATCTGGGAAGCCTTGTGCAGTATTGGGGTCTGGAACTAAAAAAGAGTGCTATTAGTCTGCTTGATACCATAGCAATAGCAGTAGCTAACGGAACAGATAGGATAATAGAAGCAATACAAGGACTGTGTAGAGCTATCAGCAACATACCTAGAAGAATAAGACAGGGCTTTGAAGCAGCTTTGCAATAA

>707010457.synR1

ATGAAAGTGATGGAGATATTGAGGAAGTGGAAACATTGGTGGATATGGGGAATCTTAGGCTTTTGGATGTTATTAATTAGTAATGTAATGGGGAACTTGTGGGTCACAGTCTATTATGGGGTACCTGTGTGGAGAGAAGCAAAAACTACTTTATTCTGTGCATCAGATGCTAAAGCATATGAGAGAGAAGTGCATAATGTCTGGGCTACACATGCCTGTGTACCCACAGACCCCAACCCACAGGAAATGAGGTTAGAAAATGTAACAGAAAATTTTAACATGTGGAAAAATGACATGGTGGATCAGATGCATGAGGATATAATCAGTTTATGGGATCAAAGCCTAAAGCCATGTGTAAAGTTGACCCCACTCTGTGTCACTTTAAGATGTGAGAATGCTACCTACAATGATACCAACTACAAGAATATCACAGATGGGGAAGTAATGAATTGCTCTTTCAATATAACCACAGAAATAAGAGATAAGAGAAGAAAAGAATCTGCACTCTTCTATAGAGTTGATATAGTACCACTTGACAACAACAGTGCTGAGTATAGATTAATAAATTGTAAAACCTCAAGCCTAACACAAGCATGCCCAAAGATCTCTTTTGACCCAATTCCTATACATTATTGTGCTCCAGCTGGTTATGCGATTCTAAAGTGTAACAATAAGACATTCAATGGAACAGGGCCATGCAATAGTGTCAGTACAGTACAGTGTACACATGGAATTAAGCCAGTGGTATCCACTCAACTACTGTTAAATGGTAGCCTAGCAGAAGAAGATATAATAATTAGATCTGAAAATCTGACAGACAATGCCAAAATAATAATAGTACATCTTAATGAACCTGTAGAAATTAGGTGTACAAGACCTGGCAATAATACAAGGAAAAGTGTGAGAATAGGGCCAGGACAAACATTCTATGCAACAGGAGAAATAATAGGAGATATAAGAAAAGCACATTGTAACATTAGTAGAAGTAAGTGGAATGAAACTTTAGAGAGGATAAGTAACAAATTAGAAGAACACTTCCCTAATAAAACAATACAATTTAACTCAAGCTCAGGAGGGGACCTAGAAATTACCACACATAGCTTTAATTGTAGAGGGGAGTTCTTCTATTGTAATACATCAAAACTGTTTAATAATAATATAACAAGCGATAATTCAAGCAACATCACAATCCCATGCAAAATAAAACAAATTATAAACATGTGGCAGAAGGTAGGAAAAGCAATGTATGCCCCTCCCATTGCAGGGAACATAACATGTACCTCGAATATCACAGGAATACTATTAACACGTGATGGAGGAAATAATAGTGACAGTGATCCAGAGACATTCAGACCTACAGGAGGAGATATGAGGGACAATTGGAGAAGTGAATTATATAAATATAAAGTAGTAGAAGTAGAACCATTAGGAATAGCACCCACTGGAGCAAAAAGGAGAGTGGTGGAGAGACAGAAAAGAGCAGTGGGAATGGGAGCTATGTTCCTTGGGTTCTTGGGAGCAGCAGGAAGCACTATGGGCGCAGCGTCAATAACGCTGACGGTACAGGCCAGACAATTATTGTCTGGTATAGTGCAACAGCAAAGCAATTTGCTGAGGGCTATAGAGGCGCAACAGCATATATTGCAACTCACAGTCTGGGGCATTAAACAGCTCCAGACAAGAATCCTGGCTATAGAAAGATACCTAAAGGATCAACAGCTCCTAGGGATTTGGGGCTGCTCTGGAAAACTCATCTGCACCACTTCTGTACCTTGGAACTCTAGTTGGAGCAATAAATCTGAATCTGAGATTTGGAATAACATGACCTGGATGCAATGGGATAAAGAAATTAGTAATTACACAAACACAATATACAGGTTGCTTGAAGAATCGCAAAACCAGCAGGAAAAGAATGAACAAGATTTACTAGCATTGGACAGTTGGAAAAATCTGTGGAGTTGGTTTAGTATAACAAATTGGCTGTGGTATATAAAAATATTCATAATGATAGTAGGAGGCCTGATAGGTTTAAGAATAATTTTTGCCATTTTGTCTATAGTGAATAGAGTTAGGCAGGGATACTCACCTTTGTCGTTTCAGACCCTTACCCCAAACCCGAGGGGACCCGACAGGCTCGGAAGAATCGAAGAAGGAGGTGGAGAGCAAGACAACGACAGATCCACTCGATTAGTGAGCGGATTCTTAGCACTTGCCTGGGACGATCTGCGGAGCCTGTTCCTCTTCAGCTACCACCGATTGAGAGACTTCACATTGATTGTAGCGAGGGTGGTGGAACTTCTGGGACGCAGCAGCCTCAGGGGACTACAGAAGGGGTGGGAAACCCTTAAGTATCTGGGAAGTCTTGTGCAGTATTGGGGTCTAGAACTAAAAAAGAGTGCTATTAGTCTGCTTGATACCATAGCAATAGCAACAGCTGAAGGAACAGATAGGATTATAGAACTAGTACAAAGAATTTGTAGGGCTATCTGCAACGTACCTACAAGAATAAGACAGGGCCTTGAAGCAGCTTTGCAATAA

>705010534.synR1

ATGAGAGTGATGGGGATACAGAAGAACTGTCAACAATGGTGGATATGGGGCATCTTAGGCTTTTGGATGTTATTGATTTGTAATGTGCAGAGCTCTTGGGTCACAGTCTACTATGGGGTACCTGTGTGGAAGGAAGCAAAAACTACTTTATTTTGTGCATCAGATGCGAAAGCATATGATGAGGAAGTACATAATGTCTGGGCTACACATGCCTGTGTACCCACAGACCCCAGCCCACAAGAAATGTTTTTGGAGAATGTAACAGAAGATTTTAACATGTGGAAAAATGACATGGTAGATCAGATGCATGAGGATATAATCAGTTTATGGGATCAAAGCCTAAAGCCATGTGTAAAGTTGACCCCACTCTGTGTCACTTTAGAGTGCACAAATGCTACATTCAATACTAGCATAAAGAAAGAAATGAGACAATGCTCTTTCAATGTAACCACAGTAGTAAGAGATAAGAAAAGGAAAGAAAATGCACTTTTTTATAAACTTGATATAGTACCACTTAATGGAAATTCTAGTGGAAATGGAAATGTTAGTGAGTATAGATTAATAAATTGTAATACCTCAACCATAACACAAGCCTGTCCAAAGGTCACTTTTGATCCAATCCCTATACATTACTGCACTCCAGCTGGTTATGCGATTCTAAAGTGTAATGATAATACATTTAATGGAACAGGGCCATGCACGAATGTTAGCACAGTACAATGTACACATGGAATTAAGCCAGTGGTATCAACTCAGCTACTGTTAAATGGCAGCAGAGCAGAAAAAGAGATAGTAATTAGATCTGAAAACATAATAAACAATGTCAAGACAATAATAGTACATCTTAATGAATCTGTAGAGATTGAGTGTACAAGGGTTGGCAATAATACAAGGAAAAGTGTGAGAATAGGACCAGGACAAACATTTTATGCAACAGGAGATATAATAGGAGACATAAGGGAAGCACATTGTAACATTAGTGAAGAAACATGGAATAAAACTCTACACATGGTAAGTGAAAAATTAAAAGAACACTTCCCTAATAAAACAATAATGTTTAACCCACACGCAGGAGGGGATCTAGAAATTACAACACATAGCTTTAACTGTAGAGGAGAATTTTTCTATTGCAATACATCAAGACTATTTAATGGTACATACATGTCTATGTCTAATAATACAAAGAATGATACAAACGCAACCATCACACTCCCATGCAGAATAAAACAAATTATAAACATGTGGCAGGGGGTAGGACGAGCAATGTATGCCCCTCCCATTGCAGGAGACATAACATGTAAATCAACTATAACAGGACTAATATTGACACGTGATGGAGGGGAAGAAAGCAAAACTAATAATACAGAGATATTTAGACCTGCAGGAGGAGACATGAGGGACAATTGGAGGAGTGAATTATATAAATATAAAGTGGTAGAAATTAAGCCATTAGGAGTAGCACCCACTGAGGCAAAAAGGAGAGTGGTGGAGAGAGAGAAAAGAGCAGTGGGAATAGGAGCTTTGTTCCTTGGGTTCTTGGGAACAGCAGGAAGCACTATGGGCGCGGCGTCAATAACGCTGACGGTACAGGCCAGACAATTATTGTCTGGTATAGTGCAACAGCAAAACAATTTGCTGAAGGCTATAGAGGCGCAACAGCATATGTTGCAACTCACGGTCTGGGGCATTAAACAGCTCCAGGCAAGAGTCCTGGCTCTAGAAAGATACCTAAGGGATCAACAGCTCCTAGGGATGTGGGGCTGCTCTGGAAAACTCATCTGCACCACTGCTGTGTATTGGAACTCTAGTTGGAGTAATAAAACTCAAGAAGAGATTTGGGAGAACATGACCTGGATGCAATGGGATAGAGAAATTAGTAATTACACAAACACAATTTACAAGTTGCTTGAGGAATCACAAAACCAGCAGGAGAAAAATGAACAAGATTTACTAACATTGGACAGTTGGAACAATCTGTGGAATTGGTTTAGCATAACAAAATGGTTGTGGTATATAAAAATATTTATAATGATAGTAGGAGGCTTGATAGGGTTAAGAATAATTTTTGCTGTGCTTTCTATAGTAAATAGAGTTAGGCAGGGATACTCACCCTTGTCATTTCAGACCCTTACCCCAAACCAGAGGGAACTCGACAGGCTCGAAAGAATCGAAGAAGGAGGTGGAGAGCAAGACAGAGACAGATCCATTCGATTAGTGAACGGATTCTTGTCACTTGCCTGGGACGACCTGCGGAGCCTGTGCCTTTTCAGCTACCACCGATTGAGAGACTTCATATTGATTGTAGTGAGAGCAGTGGAACTTCTGGGACGCAGCAGCATCAAGGGACTGCAGAGGGGGTGGGAAATCCTTAAGTATCTGGGAAGTCTTGTGCAATATTGGATTCTAGAGCTAAAAAAGAGTGCTATTAGTCTGCTTGATACCACAGCAATAGCAGTAGCTACAGGAACAGATAGGATTATAGAAGTAATACAAAGAGGGTGTAGAGCTATCTGCAACATACCTAGAAGAATAAGACAAGGCTTTGAAACCGCATTGCTATAA

>CH457

TGGAAGGGTTAATTTACTCCAAGAAAAGGCAAGACATCCTTGATTTATGGGTCTATCACACACAAGGTTACTTCCCTGATTGGCAAAACTACACACCAGGACCAGGGGTCAGATATCCACTGACCTTGGGATGGTGCTACAAGCTAGTGCCAGTTGATCCAAAGGAAGTAGAAGAGGAAACGGAAGGAGAGAACAACAGTTTGCTACACCCTATGAGCCTGCATGGAATGGAGGATGAACACAGAGAAGTATTAAAGTGGAAGTTTGACAGTATGCTAGCACGCAGACACATGGCCCGCGAGAAACATCCGGAGTATTACAAAGACTGCTGACACAGAAGGGACTTTCCGCTGGGACTTTCCACTGGGGCGTTCCGGGAGGTGTGGTCTGGGCGGGACGGGGAGTGGCCAACCCTCAGATGCTGCATATAAGCAGCTGCTTTTCGCCTGTACTGGGTCTCTCTAGGTAGACCAGATCTGAGCCTGGGAGCTCTCTGGCTATCTGGGGAACCCACTGCTTAAGCCTCAATAGAGCTTGCCTTGAGTGCTCTAAGTAGTGTGTGCCCGTCTGTTGTGTGACTCTGGTAACTAGAGATCCCTCAGACCAATTGTGGTAGTGTGGAAAATCTCTAGCAGTGGCGCCCGAACAGGGACCCGAAAGCGAAAGTAAGACCAGAGAAGATCTCTCGACGCAGGACTCGGCTTGCTGAAGTGCACTCGGCAAGAGGCGAGAGCGGCGGCTGGTGAGTACGCCAATTTTTATTTGACTAGCGGAGGCTAGAAGGAGAGAGATGGGTGCGAGTGCGTCAATATTACGAGGGGAAAAATTAGATAAATGGGAAAAAATTAGGTTAAGGCCAGGGGGAAAGAAAAGCTATATGCTAAAACACATAGTATGGGCAAGCAGGGAGCTGGAAAGATTCGCGCTTAACCCTGGTCTTTTAGAGACAGAAGAAGGCTGTAAACAAATAATGAAACAGCTACAACCAGCTCTTCAGACAGGAACAGAGGAACTTAGATCATTATACAACACAGTAGCAGTTCTCTATTGTGTACATAAAAGGATAGAAGTACGAGACACCAAGGAGGCTTTAGACAGGATAGAGGAAGAACAAAACAAAAGCCAGCAAAAAACACAGCAAGCCGAAGCGGCTGACAAGGTCAGTCAAAATTATCCTATAGTGCAGAATCTTCAAGGGCAAATGGTACACCAGGCCCTATCACCGAGAACTTTGAATGCCTGGGTAAAAGTAATAGAGGAAAAGGCTTTCAGCCCAGAGGTAATACCCATGTTCACAGCCTTATCAGAAGGAGCCACCCCACAAGATTTAAACACCATGTTAAATACAGTGGGGGGACATCAAGCAGCCATGCAAATGTTAAAAGACACCATCAATGAGGAGGCTGCAGAATGGGATAGAGTACATCCAGTACCTGCAGGGCCTGTCGCACCAGGCCAATTGAGAGACCCAAGGGGAAGTGACATAGCAGGAACTACTAGTACCCTTCAGGAACAGATAGCATGGATGACAGCCAACCCAGCTATTCCAGTAGGAGACATCTATAAAAGATGGATAATTCTGGGGTTAAATAAAATAGTAAGAATGTATAGCCCTGTCAGCATTTTGGACATAAAACAAGGGCCAAAAGAACCCTTTAGAGACTATGTAGACCGGTTCTTTAAAACTCTAAGAGCTGAACAAGCTACACAAGAGGTAAAAAATTGGATGACAGACACCTTGTTGATCCAAAATGCGAATCCAGATTGTAAGACCATTTTAAAAGCATTAGGGCCAGGGGCTACATTAGAAGAAATGATGACAGCATGTCAGGGAGTGGGAGGACCTAGCCACAAAGCAAGAGTGTTGGCTGAGGCAATGAGTCAAGCAAACAATGCAAACATAATGATGCAGAGAAGCAATTTTAAAGGCACTAAAAGAATTATTAAATGTTTCAATTGTGGCAAAGAGGGACACATAGCCAGAAATTGCAGGGCTCCTAGGAAAAAGGGCTGTTGGAAATGTGGGAAAGAAGGACATCAGATGAAAGATTGTACTGAAAGACAGGCTAATTTTTTAGGGAAAATTTGGCCTTCCCACAAGGGGAGGCCGGGAAATTTCCTTCAGAACAGAACAGAGCCAACAGCCCCACCAGCAGAGAGCTTCGAGTTCGAGGAGACAACCCACCCTCCGAAGCAGGAGCAGAAAAACAGGGAAACCTTAACTTCCCTCAAATCACTCTTTGGCAGCGACCCCTTGTCTCAATAAAAGTAGGGGGCCAGATAAAGGAAGCTCTCTTAGATACAGGAGCAGATGATACAGTATTAGAAGATATAAATTTGCCAGGAAAATGGAAACCAAAAATGATAGGAGGAATAGGAGGCTTTATCAAAGTAAGACAGTATGATCAAATAGTTATAGAAATTTGTGGAAAAAAGGCTATAGGTTCAGTATTAGTGGGACCAACACCGGTCAACATAATTGGAAGAAATATGTTGACTCAGCTTGGATGCACACTAAATTTTCCAATTAGTCCCATTGAGACTGTACCAGTGAAATTAAAGCCAGGAATGGATGGCCCAAAAGTTAAACAATGGCCATTGACTGAGGAAAAAATAAAAGCATTAACAGCAATTTGTGAGGATATGGAAAAGGAAGGAAAAATTACAAAAATTGGGCCTGAAAATCCCTATAACACTCCAGTATTCGCCATAAAAAAGAAAGATAGTACTAAGTGGAGAAAATTAGTAGATTTTAGAGAACTCAATAAAAGAACTCAAGACTTTTGGGAAGTTCAATTAGGGATCCCACACCCAGCAGGGTTGAAGAAGAAAAAATCAGTGACAGTACTAGATGTGGGGGATGCATACTTTTCAGTTCCTCTAGATGAAGATTTCAGGAAGTATACTGCATTCACCATACCTAGTATAAACAATGAAACACCAGGGATTAGATACCAATATAATGTGCTTCCACAGGGATGGAAAGGATCGCCAGCAATATTCCAGAGTAGCATGACAAAAATCTTAGAGCCCTTCAGGGTAAGAAACCCAGACATAGTTATCTATCAATATATGGATGACTTGTATGTAGGATCTGATTTAGAAATAGGGCAACATAGAGCAAAAATAGAGGAATTAAGAGAACATTTGTTGAAATGGGGATTTACCACACCAGACAAGAAACATCAGAAAGAACCCCCATTTCTTTGGATGGGGTATGAACTCCATCCTGACAAATGGACAGTACAGCCTATAAAGCTACCAGAAAAGAATAGCTGGACTGTCAATGATATACAGAAGTTAGTGGGAAAATTAAACTGGGCAAGTCAGATTTACTCAGGAATCAAAGTAAAGCAACTTTGTAAACTCCTTAGGGGGACCAAAGCATTAACAGACATAGTACCACTAACTGAAGAAGCAGAATTAGAATTGGAGGAGAACAGGGAAATTCTAAGAGAACCAGTACATGGAGCATATTATGACCCATCAAAAGACTTAATAGCTGAAATACAGAAACAGGGGCATGATCAATGGACATATCAAATTTACCAAGAACCTTTCAAAAATCTGAAGACAGGAAAATATGCAAAAATGAGGTCCGCCCATACTAATGATGTAAAGCAGTTAACAGAAGCAGTGCAGAAAATAGCCATGGAAAGCATAGTAATATGGGGGAAGACCCCTAAATTTAGACTACCCATCCAAAAAGAAACATGGGAGACATGGTGGACAGACTATTGGCAAGCCACCTGGATTCCTGAGTGGGAGTTTGTTAATACCCCTCCCCTAGTAAAATTATGGTACCAGCTGGAGAAAGAACCCATAGTAGGAGCAGAGACCTTCTATGTAGATGGAGCAGCTAATAGGGAAACTAAAATAGGAAAAGCAGGGTATGTTACTGACAGAGGAAGACAGAAAATTGTTTCTCTAACAGAAACAACAAATCAGAAGACTGAATTACAAGCAATTCAGCTAGCCTTGCAGGACTCAGGATCAGAAGTAAACATAGTAACAGATTCACAGTATGCATTAGGAATCATTCAAGCACATCCAGATAAGAGTGAATCAGAGATAGTTAATCAAATAATAGAACAGTTAATAAACAAGGAAAGAGTCTATCTGTCATGGGTACCAGCACATAAAGGAATTGGAGGAAATGAACAAGTAGATAAATTAGTAAGTAATGGAATCAGGAAAGTACTGTTTCTAGATGGAATAGATAAGGCTCAGGAAGAGCATGAAAAGTATCACAGCAATTGGAGAGCAATGGCTAGTGAATTTAATCTGCCACCCATAGTAGCTAAAGAAATAGTAGCTAGCTGTGATAAATGTCAGCAAAAAGGGGAAGCAATACATGGACAAGTCGACTGTAGTCCAGGGATATGGCAATTAGACTGTACACATTTAGAAGGAAAAATTATCCTGGTAGCAGTCCATGTAGCCAGTGGCTACATAGAAGCAGAGGTTATTCCAGAAGAAACAGGACAAGAAACAGCATACTATATACTAAAATTAGCAGGAAGATGGCCAGTTAAAGTAATACACACAGACAATGGCAGAAATTTCACCAGTAATACAGTTAAGGCAGCCTGTTGGTGGGCGGGTATCAAACAAGAATTTGGTATTCCATACAATCCCCAAAGTCAGGGAGTAGTGGAATCCATGAATAAAGAACTAAAGAAGATCATAGGGCAGGTAAGAGATCAAGCTGAGCACCTTAAGACAGCAGTACAAATGGCAGTATTCATTCACAATTTTAAAAGAAAAGGGGGGATTGGGGATTATACTGCAGGGGAAAGAATAATAGACATAATAGCAACAGACATACAAACTAAAGAATTACAAAAACAAATTATAAAAATTCAAAATTTTCGGGTTTATTACAGAGACAGCAGAGACCCTATTTGGAAAGGACCAGCCAAACTACTATGGAAAGGTGAAGGGGCAGTAGTAATACAAGACAAAAGTGACATAAAGGTAGTACCAAGGAGAAAAGTAAAAATTATTAAACACTATGGAAAACAGATGGCAGGTGCTGATTGTGTGGCAGATAGACAGGATGAGGATCAGAACATGGAATAGTTTAGTAAAGCACCATATGTATGTTTCAAGGAGAGCTAACGGATGGTACTACAGACATCATTATGAAAGCAGACATCCAAAAGTAAGTTCAGAAGTACATATCCCATTAGGGGATGCTAGATTAGTAATAAAAACATATTGGGGGTTGCAGACAGGAGAAAGAGAGTGGCATTTGGGTCATGGAGTCTCCATAGAATGGAGACTTAGAAGATATAGCACACAAGTAGACCCTGGCCTGGCAGACCAACTAATTCATATGCATTATTTTGATTGTTTTGCAGACTCTGCCATAAGAAAAGCCATATTAGGACACATAGTTACTCCTAGGTGTGACTATCAAGCAGGCCATAATCAGGTAGGATCTCTGCAATACTTGGCACTGACAGCATTAGTAAAACCAAAAAAGATAAAGCCACCTCTGCCTAGTGTTAGAAAATTAGTAGAGGATAGATGGAACAACCCCCAGAAGACCAGGGGCCGCAGAGGGAACCACATAATGAATGGGCACTAGAGATTCTAGAGGAACTCAAGCAGGAAGCTGTCAGACACTTTCCTAGGCCATGGCTACATGCCTTAGGACAACAAATCTATAACACCTATGGGGATACTTGGACTGGAGTTGAGGCCATAATAAGAATACTGCAACAACTACTGTTTATTCATTTCAGAATTGGGTGCCAACATAGCAGAATAGGCATTGTGCGGCAGAGAAGAGCAAGAAATGGAGCAAGTAGATCCTAAACTAGAGCCCTGGAACCATCCAGGAAGCCAGCCTAAAACTGCTTGTAATACATGTTTCTGCAAACGCTGTAGCTATCACTGTTTAGTTTGCTTTCAGACAAAAGGCTTAGGCATTTCCTATGGCAGGAAGAAGCGGAGAAAGCGACGAAGCGCTCCTCCAAGCAGTGAGGATCATCAAAATCCTATATCAAAGCAGTAAGTACCACATAATAACACATAGTATATGTAATGTTGGATTTACTAGCAAGAGTAGATTATAGAATAGGAGTAGCAGCCTTGTTAATAGCACTAATCATAGCAATAGTTGTGTGGACCATAGTATATATAGAATATAAGAAATTGTTACGGCAAAAAAGGATAGACTGGTTAATTAAAAGAATTAGGGAAAGAGCAGAAGACAGTGGCAATGAAAGTGATGGAGATATTGAGGAAGTGGAAACATTGGTGGATATGGGGAATCTTAGGCTTTTGGATGTTATTAATTAGTAATGTAATGGGGAACTTGTGGGTCACAGTCTATTATGGGGTACCTGTGTGGAGAGAAGCAAAAACTACTTTATTCTGTGCATCAGATGCTAAAGCATATGAGAGAGAAGTGCATAATGTCTGGGCTACACATGCCTGTGTACCCACAGACCCCAACCCACAGGAAATGAGGTTAGAAAATGTAACAGAAAATTTTAACATGTGGAAAAATGACATGGTGGATCAGATGCATGAGGATATAATCAGTTTATGGGATCAAAGCCTAAAGCCATGTGTAAAGTTGACCCCACTCTGTGTCACTTTAAGATGTGAGAATGCTACCTACAATGATACCAACTACAAGAATATCACAGATGGGGAAGTAATGAATTGCTCTTTCAATATAACCACAGAAATAAGAGATAAGAGAAGAAAAGAATCTGCACTCTTCTATAGAGTTGATATAGTACCACTTGACAACAACAGTGCTGAGTATAGATTAATAAATTGTAAAACCTCAAGCCTAACACAAGCATGCCCAAAGATCTCTTTTGACCCAATTCCTATACATTATTGTGCTCCAGCTGGTTATGCGATTCTAAAGTGTAACAATAAGACATTCAATGGAACAGGGCCATGCAATAGTGTCAGTACAGTACAGTGTACACATGGAATTAAGCCAGTGGTATCCACTCAACTACTGTTAAATGGTAGCCTAGCAGAAGAAGATATAATAATTAGATCTGAAAATCTGACAGACAATGCCAAAATAATAATAGTACATCTTAATGAACCTGTAGAAATTAGGTGTACAAGACCTGGCAATAATACAAGGAAAAGTGTGAGAATAGGGCCAGGACAAACATTCTATGCAACAGGAGAAATAATAGGAGATATAAGAAAAGCACATTGTAACATTAGTAGAAGTAAGTGGAATGAAACTTTAGAGAGGATAAGTAACAAATTAGAAGAACACTTCCCTAATAAAACAATACAATTTAACTCAAGCTCAGGAGGGGACCTAGAAATTACCACACATAGCTTTAATTGTAGAGGGGAGTTCTTCTATTGTAATACATCAAAACTGTTTAATAATAATATAACAAGCGATAATTCAAGCAACATCACAATCCCATGCAAAATAAAACAAATTATAAACATGTGGCAGAAGGTAGGAAAAGCAATGTATGCCCCTCCCATTGCAGGGAACATAACATGTACCTCGAATATCACAGGAATACTATTAACACGTGATGGAGGAAATAATAGTGACAGTGATCCAGAGACATTCAGACCTACAGGAGGAGATATGAGGGACAATTGGAGAAGTGAATTATATAAATATAAAGTAGTAGAAGTAGAACCATTAGGAATAGCACCCACTGGAGCAAAAAGGAGAGTGGTGGAGAGACAGAAAAGAGCAGTGGGAATGGGAGCTATGTTCCTTGGGTTCTTGGGAGCAGCAGGAAGCACTATGGGCGCAGCGTCAATAACGCTGACGGTACAGGCCAGACAATTATTGTCTGGTATAGTGCAACAGCAAAGCAATTTGCTGAGGGCTATAGAGGCGCAACAGCATATATTGCAACTCACAGTCTGGGGCATTAAACAGCTCCAGACAAGAATCCTGGCTATAGAAAGATACCTAAAGGATCAACAGCTCCTAGGGATTTGGGGCTGCTCTGGAAAACTCATCTGCACCACTTCTGTACCTTGGAACTCTAGTTGGAGCAATAAATCTGAATCTGAGATTTGGAATAACATGACCTGGATGCAATGGGATAAAGAAATTAGTAATTACACAAACACAATATACAGGTTGCTTGAAGAATCGCAAAACCAGCAGGAAAAGAATGAACAAGATTTACTAGCATTGGACAGTTGGAAAAATCTGTGGAGTTGGTTTAGTATAACAAATTGGCTGTGGTATATAAAAATATTCATAATGATAGTAGGAGGCCTGATAGGTTTAAGAATAATTTTTGCCATTTTGTCTATAGTGAATAGAGTTAGGCAGGGATACTCACCTTTGTCGTTTCAGACCCTTACCCCAAACCCGAGGGGACCCGACAGGCTCGGAAGAATCGAAGAAGGAGGTGGAGAGCAAGACAACGACAGATCCACTCGATTAGTGAGCGGATTCTTAGCACTTGCCTGGGACGATCTGCGGAGCCTGTTCCTCTTCAGCTACCACCGATTGAGAGACTTCACATTGATTGTAGCGAGGGTGGTGGAACTTCTGGGACGCAGCAGCCTCAGGGGACTACAGAAGGGGTGGGAAACCCTTAAGTATCTGGGAAGTCTTGTGCAGTATTGGGGTCTAGAACTAAAAAAGAGTGCTATTAGTCTGCTTGATACCATAGCAATAGCAACAGCTGAAGGAACAGATAGGATTATAGAACTAGTACAAAGAATTTGTAGGGCTATCTGCAACGTACCTACAAGAATAAGACAGGGCCTTGAAGCAGCTTTGCAATAACATGGGGGGCAAGTGGTCAAAATGTAGTATGGTGGGATGGCCTGAGGTAAGAGAAAGAATAAGGAGAACTGCACCAGCAGCAGAGGGAGTAGGAGCAGCATCTCGAGACCTAGATAGGCATGGAGCAATTACAAGCAGCAACACAGCCACCACTAATGCTGCTTGTGCCTGGCTGGAAGCACAAGAGGAGGCAGAAGAAGTAGGCTTTCCAGTTAGACCTCAGGTGCCTTTAAGACCAATGACTTATAAGGGAGCAGTCGATCTCAGCTTCTTTTTAAAAGAAAAGGGGGGACTGGAAGGGTTAATTTACTCCAAGAAAAGGCAAGACATCCTTGATTTATGGGTCTATCACACACAAGGTTACTTCCCTGATTGGCAAAACTACACACCAGGACCAGGGGTCAGATATCCACTGACCTTGGGATGGTGCTACAAGCTAGTGCCAGTTGATCCAAAGGAAGTAGAAGAGGAAACGGAAGGAGAGAACAACAGTTTGCTACACCCTATGAGCCTGCATGGAATGGAGGATGAACACAGAGAAGTATTAAAGTGGAAGTTTGACAGTATGCTAGCACGCAGACACATGGCCCGCGAGAAACATCCGGAGTATTACAAAGACTGCTGACACAGAAGGGACTTTCCGCTGGGACTTTCCACTGGGGCGTTCCGGGAGGTGTGGTCTGGGCGGGACGGGGAGTGGCCAACCCTCAGATGCTGCATATAAGCAGCTGCTTTTCGCCTGTACTGGGTCTCTCTAGGTAGACCAGATCTGAGCCTGGGAGCTCTCTGGCTATCTGGGGAACCCACTGCTTAAGCCTCAATAGAGCTTGCCTTGAGTGCTCTAAGTAGTGTGTGCCCGTCTGTTGTGTGACTCTGGTAACTAGAGATCCCTCAGACCAATTGTGGTAGTGTGGAAAATCTCTAGCAAGCTT

>CH534

TGGAAGGGTTAATTTACTCCAGGAAAAGGCAAGAGATCCTTGATTTGTGGGTCCATAACACACAAGGCTTCTTCCCTGATTGGCAAAACTACACACCAGGACCAGGGGTCAGATATCCACTGACCTTTGGATGGTGCTTCAAGCTAGTGCCAGTCGACCCAAGGGAAGTGGAAGAGGCCAATGAAGGAGAAAACAACTGTTTGCTGCACCCTATGAGCCAGCATGGAATGGAGGATGAAGACAGAGAAGTCTTAATGTGGAAGTTTGACATTAGCCTAGCACACAGACACATGGCCCGCGAGATACATCCGGAGTATTACAAAGACTGCTGACACAGAAGGGACTTTCCGCGCTGGGACTTTCCACTGGGCGTTCCGGGAGGTGTGGTCTGGGCGGGACGGGGAGTGGTCAACCCTCAGATGCTGCATATAAGCAGCTGCTTTTCGCCTGTACTGGGTCTCTCTAGGTAGACCAGATCTGAGCCCGGGAGCTCTCTGGCTATCTAGGGAACCCACTGCTTAAGCCTCAATAAAGCTTGCCTTGAGTGCTTGAAGTAGTGTGTGCCCGTCTGTTGTGTGACTCTGGTAACTAGAGATCCCTCAGACCCTTGTTGGTAGTGTGGAAAATCTCTAGCAGTGGCGCCCGAACAGGGACCCGATAGCGAAAGTGAAACCAGAGGAGATCTCTCGACGCAGGACTCGGCTTGCTGAAGTGCACACGGCAAGAGGCGAGAGCGGCGACTGGTGAGTACGCCAAATTTTATTTGACTAGCGGAGGCTAGAAGGAGAGAGATGGGTGCGAGAGCGTCAATATTAAGAGGCGGAAAATTAGATAGATGGGAAAAAATTAGGTTAAGGCCAGGGGGAAAGAAACATTATATGATAAAACACTTAGTATGGGCAAGCAGGGAGCTGGAAAGATTTGCACTTAACCCTGGCCTTTTAGAAACATCAGCAGGCTGTAGACAAATAATGAAACAGCTACAACCAGCTCTCCAGACAGGAACAGAGGAACTTAGATCATTATTTAACACAGTAGCAACTCTCTATTGTGTACATGAGGAGATAGATGTACGAGACACCAAAGAAGCCCTAGACAGGATAGAGGAAGAACAAAACAAAAGTCAGCAAGCAGCAGCGGCTGACAAAGGAAAGATCAGTCAAAATTATCCAATAGTGCAGAATCTCCAAGGGCAAATGGTACACCAGCCCATATCACCAAGGACTTTAAATGCATGGGTAAAGGTAGTAGAGGAGAAGGCTTTCAGCCCAGAAGTAATCCCCATGTTTACAGCATTATCAGAAGGAGCCACCCCACAAGATTTAAACACCATGTTAAATACAGTGGGGGGACATCAAGCAGCCATGCAAATGTTAAAAGAGACCATCAATGATGAGGCTGCAGAATGGGATAGACTACATCCAGTCCATGCAGGACCTGTCGCACCAGGCCAGATGAGAGAACCAAGGGGAAGTGACATAGCAGGAACTTCTAGTACCCTTCAGGAACAAATAGCATGGATGACAAGTAACCCACCTATCCCAGTGGGAGACATCTATAAAAGATGGATAATTCTGGGGCTAAATAAAATAGTAAGAATGTATAGCCCTGTTAGCATTTTGGACATAAAACAAGGGCCAAAAGAACCCTTTAGAGATTATGTGGATCGGTTCTTTAAAACTTTAAGAGCTGAACAATCCACACAAGAGGTAAAAAATTGGATGACAGAGACCTTGTTGGTCCAAAATGCGAACCCAGATTGTAAAGGCATTTTAAGAGCATTAGGACCACATGCTACAATAGAAGAAATGATGACAGCATGTCAGGGAGTGGGAGGACCTAGCCACAAAGCAAGAGTTTTGGCTGAGGCAATGAGCCAAGTAAACAATACAAACATAATGATGCAAAAAAGCAATTTTAAAGGCCCTAAAAGAATTATCAAATGTTTCAACTGTGGCAAGGAAGGGCACATAGCCAAAAATTGCAGAGCCCCTAGAAAAAGAGGCTGTTGGAAATGTGGAAAGGAAGGACACCAAATGAAAGACTGTACTGAGAGACAGGCTAATTTTTTAGGGAAAATTTGGCCTTCCCACAAGGGGAGGCCAGGGAATTTCCTTCAGAGCAGGCCAGAACCAACAGCCCCACCAGCAGAGCCAACAGCCCCACCAGCAGAGAGTTTCAGGTTCGAGGGGACAACCCCTGCTCCGAGGCAGGAGACGAAGGACAGGGAACCTTTAACTTCCCTCAAATCACTCTTTGGCAACGACCCCTTGTCTCAATAAAAATAGGGGGCCAGATAAAGGAGGCTCTCTTAGATACAGGAGCAGATGATACAGTATTAGAAGAAATAAATTTGCCAGGAAAATGGAAACCAAAAATGATAGGAGGAATTGGGGGCTTTATCAAAGTGAGACAGTATGAACAAGTACTTATAGAAATTTGTGGAAAAAAGGCTATAGGTGCAGTATTAGTGGGACCTACACCTATCAACATAATAGGAAGAAATATGTTGACTCAGCTTGGATGCACACTAAATTTTCCAATTAGTCCCATTGAAACTGTACCAGTAAAATTAAAGCCAGGAATGGATGGCCCAAAAGTTAAACAATGGCCATTGACAGAAGAGAAAATAAAAGCATTAACAGCAATATGTGAAGAAATGGAGAAGGAAGGAAAAATTACAAAAATTGGGCCTGAAAATCCATATAACACTCCAGTATTTGCCATAAAAAAGAAGGACAGTACTAAGTGGAGAAAATTAGTAGATTTCAGGGAACTCAATAAAAGGACTCAAGACTTTTGGGAAGTTCAATTAGGAATACCACACCCAGCAGGGTTAAAAAAGAAAAAATCAGTGACAGTGCTGGATGTGGGGGATGCATATTTTTCAGTTCCTCTAGATGAAAACTTCAGGAAATATACTGCATTCACCATACCCAGCATAAATAATGAAACACCAGGGATTAGATACCAATATAATGTGCTTCCACAAGGATGGAAAGGATCACCAGCAATATTTCAGAGTAGCATGACAAGAATCCTAGAGCCCTTTAGGATGAAAAATCCAGAAATGGTCATTTATCAATATATGGATGACTTGTATGTAGGATCTGACTTAGAAATAGGGCAACATAGAGCAAAAATAGAGGAGTTAAGGGAACATTTATTGAAATGGGGATTTACCACACCAGACAAGAAACATCAAAAGGAACCCCCATTTCTTTGGATGGGGTATGAACTCCATCCTGACAAATGGACAGTACAGCCTATACTGTTGCCAGAAAAGGATAGCTGGACTGTCAATGACATACAGAAGTTAGTGGGAAAATTAAACTGGGCCAGTCAGATTTACCCAGGGATTAAGGTAAGGCAGCTTTGTAAACTCCTTAGGGGGACCAAAGCACTAACAGACATAGTACCACTAACTGAAGAAGCAGAATTAGAATTGGCAGAGAACAGGGAAATTCTAAAAGAACCAGTACATGGGGTATACTATGACCCTTCAAAAGACTTAATAGCTGAAATACAGAAACAGGGGCTTGACCAATGGACATATCAAATTTACCAAGAACCATTTAAAAATCTGAAAACAGGGAAGTATGCAAAAATGAGAACTGCCCACACTAATGATGTAAAGCAGTTAGCAGAGGCAGTGCAAAAAATAGCTATGGAAAGCATAGTAATATGGGGAAAGACTCCTAAATTTAGACTACCCATCCAAAGGGAAACATGGGAAATATGGTGGACAGACTATTGGCAAGCCACCTGGATTCCTGAGTGGGAGTTTGTTAATACCCCTCCTCTAGTAAAATTATGGTACCAGCTAGAGAAAGAACCCATAGCAGGAGCAGAAACTTTCTATGTAGATGGAGCAGCTAATAGGGAAACTAAATTAGGAAAAGCAGGGTATGTTACTGACAGAGGAAGACAGAAAATTGTTCCCCTAACAGAAACCACAAATCAGAAGGCTGAGTTACAAGCAATTCAGCTAGCTTTGCAGGATTCAGGATCAGAAGTAAACATAGTAACAGACTCACAGTATGCATTAGGAATCATTCAAGCACAACCAGATAAGAGTGAATCAGAGATAGTTAACCAAATAATAGAACAGTTAATAAACAAGGAAAGAACCTACCTGTCATGGGTACCAGCACATAAAGGAATTGGAGGAAATGAACAAGTAGATAAATTAGTAAGTAAGGGAATCAGGAAAGTGCTATTTCTAGATGGAATAGACAGGGCTCAAGAAGAGCATGAAAAATATCACAATAACTGGAGAGCAATGGCCAGTGACTTTAATCTGCCACCTATAGTAGCAAAAGAAATAGTAGCTAGCTGTGATAAGTGCCAGCTAAAAGGGGAAGCCATGCATGGACAAGTAGACTGTAGCCCAGGGATATGGCAATTAGACTGTACACATCTAGAGGGAAAAGTTATCTTGGTAGCAGTCCATGTAGCCAGTGGCTACATGGAAGCAGAGGTTATCCCAGCAGAAACAGGACAAGACACAGCATACTTTATACTAAAATTAGCAGGAAGATGGCCAGTCAAAGTAATACATACAGACAATGGCAGTAATTTCACCAGTGCTGCAGTTAAGGCAGCCTGTTGGTGGGCAGGTATCCAACAGGAATTTGGAATTCCCTACAATCCCCAAAGTCAGGGAGTAGTAGAATCCATGAATAAAGAATTAAAGAAAATCATAGGGCAGGTAAGAGATCAAGCTGAGCACCTTAAAACAGCAGTACAAATGGCAGTATTCATTCACAATTTTAAAAGAAAAGGGGGGATTGGGGGGTACAGTGCAGGGGAGAGAATAATAGACATAATAGCAACAGACATACAAACTAAAGAATTACAAAAACAAATTATAAAAATTCAAAATTTTCGGGTTTATTACAGAGACAGCAGAGACCCTATTTGGAAAGGACCAGCCAAACTACTCTGGAAAGGTGAAGGGGCAATAGTAATACAAGATAATAGTGACATAAAGGTAGTGCCAAGGAGGAAAGCAAAAATTATTAAGGATTATGGAAAACAGATGGCAGGTGCTGATTGTGTGGCAGATAGACAGGATGAAGATTAGAACATGGAATAGTTTAGTAAAGCACCATATGTATATATCAAAGAAAGCTAGTGGGTGGTTCTACAAACATCATTATGAGAGCAGACATCCAAAAGTAAGTTCAGAAGTACATATTCCATTAGGGGATGCTAAATTAGTAGTAAAAACATATTGGGGCTTGCAGACAGGAGAAAGAGCCTGGCATTTGGGTCATGGAGCTTCCATAGAATGGAGGCTGAGAGAATATAGCACACAAGTAGATCCTGGCCTGGCAGACCAACTAATTCATATGCATTATTTTGATTGTTTTGCAGACTCTGCAATAAGAAAAGCCCTATTAGGACATATAGTTAGCCCTAGGTGTGAATATCAAGCAGGACATAACAAGGTAGGATCTCTACAATATTTGGCACTGACAGCATTAATAAAACCAAAAAAGACAAAACCACCTCTGCCTAGTGTTAGTAAATTAGTAGAGGATAGATGGAACAAGCCCCAGAAGACCAGGGGCCGCAGAGGGAACCACACAATGAATGGACATTAGAGCTTTTAGAAGAACTCAAGCAGGAAGCTGTCAGACATTTTCCTAGACCGTGGCTTCATAGTTTAGGACAATATATATATGAAAACTATGGGGATACTTGGACAGGAGTCGAAGTGCTAATAAGAATTCTGCAACAATTATTGTTTATTCATTTCAGAATTGGGTGCCAACATAGTAGAATAGGCATTTTGCGACAGAGAAGAGCAAGAAATGGAGCCAATAGATCCTAACCTAGAACCCTGGAACCATCCAGGAAGCCAGCCTAAAACTGCTTGTAATAAATGTTATTGCAAAAAATGCAGCTACCATTGTCTAGTTTGCTTTCAGACAAAAGGCTTAGGCATTTCCTATGGCAGGAAGAAGCGGAGACAACGACGAAGCACTCCTTCAAGCAGTAAGGATCATCAAAATCCTCTATCAAAGCAGTAAGTACCCAATAGTAGATGTAATGTTAAACTTAGATTATAGAGTAGGGATAGGAGCATTGATAGTAGCACTAAATATAGCAATAGTAGTGTGGATCATAGTATATATAGAATATAGGAAACTGGTAAGACAAAGAAGAATAGACTGGTTAGTTAAAAGAATTAGGGAAAGAGCAGAAGATAGTGGCAATGAGAGTGATGGGGATACAGAAGAACTGTCAACAATGGTGGATATGGGGCATCTTAGGCTTTTGGATGTTATTGATTTGTAATGTGCAGAGCTCTTGGGTCACAGTCTACTATGGGGTACCTGTGTGGAAGGAAGCAAAAACTACTTTATTTTGTGCATCAGATGCGAAAGCATATGATGAGGAAGTACATAATGTCTGGGCTACACATGCCTGTGTACCCACAGACCCCAGCCCACAAGAAATGTTTTTGGAGAATGTAACAGAAGATTTTAACATGTGGAAAAATGACATGGTAGATCAGATGCATGAGGATATAATCAGTTTATGGGATCAAAGCCTAAAGCCATGTGTAAAGTTGACCCCACTCTGTGTCACTTTAGAGTGCACAAATGCTACATTCAATACTAGCATAAAGAAAGAAATGAGACAATGCTCTTTCAATGTAACCACAGTAGTAAGAGATAAGAAAAGGAAAGAAAATGCACTTTTTTATAAACTTGATATAGTACCACTTAATGGAAATTCTAGTGGAAATGGAAATGTTAGTGAGTATAGATTAATAAATTGTAATACCTCAACCATAACACAAGCCTGTCCAAAGGTCACTTTTGATCCAATCCCTATACATTACTGCACTCCAGCTGGTTATGCGATTCTAAAGTGTAATGATAATACATTTAATGGAACAGGGCCATGCACGAATGTTAGCACAGTACAATGTACACATGGAATTAAGCCAGTGGTATCAACTCAGCTACTGTTAAATGGCAGCAGAGCAGAAAAAGAGATAGTAATTAGATCTGAAAACATAATAAACAATGTCAAGACAATAATAGTACATCTTAATGAATCTGTAGAGATTGAGTGTACAAGGGTTGGCAATAATACAAGGAAAAGTGTGAGAATAGGACCAGGACAAACATTTTATGCAACAGGAGATATAATAGGAGACATAAGGGAAGCACATTGTAACATTAGTGAAGAAACATGGAATAAAACTCTACACATGGTAAGTGAAAAATTAAAAGAACACTTCCCTAATAAAACAATAATGTTTAACCCACACGCAGGAGGGGATCTAGAAATTACAACACATAGCTTTAACTGTAGAGGAGAATTTTTCTATTGCAATACATCAAGACTATTTAATGGTACATACATGTCTATGTCTAATAATACAAAGAATGATACAAACGCAACCATCACACTCCCATGCAGAATAAAACAAATTATAAACATGTGGCAGGGGGTAGGACGAGCAATGTATGCCCCTCCCATTGCAGGAGACATAACATGTAAATCAACTATAACAGGACTAATATTGACACGTGATGGAGGGGAAGAAAGCAAAACTAATAATACAGAGATATTTAGACCTGCAGGAGGAGACATGAGGGACAATTGGAGGAGTGAATTATATAAATATAAAGTGGTAGAAATTAAGCCATTAGGAGTAGCACCCACTGAGGCAAAAAGGAGAGTGGTGGAGAGAGAGAAAAGAGCAGTGGGAATAGGAGCTTTGTTCCTTGGGTTCTTGGGAACAGCAGGAAGCACTATGGGCGCGGCGTCAATAACGCTGACGGTACAGGCCAGACAATTATTGTCTGGTATAGTGCAACAGCAAAACAATTTGCTGAAGGCTATAGAGGCGCAACAGCATATGTTGCAACTCACGGTCTGGGGCATTAAACAGCTCCAGGCAAGAGTCCTGGCTCTAGAAAGATACCTAAGGGATCAACAGCTCCTAGGGATGTGGGGCTGCTCTGGAAAACTCATCTGCACCACTGCTGTGTATTGGAACTCTAGTTGGAGTAATAAAACTCAAGAAGAGATTTGGGAGAACATGACCTGGATGCAATGGGATAGAGAAATTAGTAATTACACAAACACAATTTACAAGTTGCTTGAGGAATCACAAAACCAGCAGGAGAAAAATGAACAAGATTTACTAACATTGGACAGTTGGAACAATCTGTGGAATTGGTTTAGCATAACAAAATGGTTGTGGTATATAAAAATATTTATAATGATAGTAGGAGGCTTGATAGGGTTAAGAATAATTTTTGCTGTGCTTTCTATAGTAAATAGAGTTAGGCAGGGATACTCACCCTTGTCATTTCAGACCCTTACCCCAAACCAGAGGGAACTCGACAGGCTCGAAAGAATCGAAGAAGGAGGTGGAGAGCAAGACAGAGACAGATCCATTCGATTAGTGAACGGATTCTTGTCACTTGCCTGGGACGACCTGCGGAGCCTGTGCCTTTTCAGCTACCACCGATTGAGAGACTTCATATTGATTGTAGTGAGAGCAGTGGAACTTCTGGGACGCAGCAGCATCAAGGGACTGCAGAGGGGGTGGGAAATCCTTAAGTATCTGGGAAGTCTTGTGCAATATTGGATTCTAGAGCTAAAAAAGAGTGCTATTAGTCTGCTTGATACCACAGCAATAGCAGTAGCTACAGGAACAGATAGGATTATAGAAGTAATACAAAGAGGGTGTAGAGCTATCTGCAACATACCTAGAAGAATAAGACAAGGCTTTGAAACCGCATTGCTATAACATGGGGGGCAAATGGTCAAAAAGCAGTATAGTTGGATGGCCTGAGGTCAGAGAAAGAATAAGACGAACTAGGCCAGCAGCAGAGGGAGAGAAAACAGCAGCAGAGGGAGTAGGAACAGCGTCTCAAGACCTAGATAAACATGGGGCACTTACAACCAGCAACACAGCCAGAAACAATGATGCTTGTGCCTGGCTAGAAGCGCAGGAAGAAGACGGGGATGTAGGCTTTCCAGTCAGACCTCAGGTGCCTTTAAGACCAATGACCTTTAAGGCAGCATTTGATCTCAGCTTCTTTTTAAAAGAAAAGGGGGGACTGGAAGGGTTAATTTACTCCAGGAAAAGGCAAGAGATCCTTGATTTGTGGGTCCATAACACACAAGGCTTCTTCCCTGATTGGCAAAACTACACACCAGGACCAGGGGTCAGATATCCACTGACCTTTGGATGGTGCTTCAAGCTAGTGCCAGTCGACCCAAGGGAAGTGGAAGAGGCCAATGAAGGAGAAAACAACTGTTTGCTGCACCCTATGAGCCAGCATGGAATGGAGGATGAAGACAGAGAAGTCTTAATGTGGAAGTTTGACATTAGCCTAGCACACAGACACATGGCCCGCGAGATACATCCGGAGTATTACAAAGACTGCTGACACAGAAGGGACTTTCCGCGCTGGGACTTTCCACTGGGCGTTCCGGGAGGTGTGGTCTGGGCGGGACGGGGAGTGGTCAACCCTCAGATGCTGCATATAAGCAGCTGCTTTTCGCCTGTACTGGGTCTCTCTAGGTAGACCAGATCTGAGCCCGGGAGCTCTCTGGCTATCTAGGGAACCCACTGCTTAAGCCTCAATAAAGCTTGCCTTGAGTGCTTGAAGTAGTGTGTGCCCGTCTGTTGTGTGACTCTGGTAACTAGAGATCCCTCAGACCCTTGTTGGTAGTGTGGAAAATCTCTAGC

>CH432

CTGGAAGGGTTAATTTACTCCAAGAAGAGGCAAGAAATCCTTGATCTGTGGGTTTATCACACACAAGGCTTCTTCCCTGATTGGCAAAACTACACACCAGGACCAGGGGTCAGATACCCACTGACCTTTGGATGGTGCTACAAGCTAGTGCCAGTAGACCCAAGTGAAGTGGAAGAAGCCAGCGGAGGAGAAGACAACTGCCTGTTACACCCTATGAACCTGCATGGAATAGAGGATGAACACAGAGAAGTATTAAAATGGAAGTTTGACAGTCAACTAGCACGCAGACACCTGGCCCGTGAGAAACATCCGGAGTTTTACAAAGACTGCTGACACAGAAAGGACTTTGCTGACACAGAAGGGACTTTCCGCTGGGACTTTCCACTGGGGCGTTCCAGGGGGAGTGGTCTGGGCGGGACGGGGAGTGGTCAACCCTCAGATGCTGCATATAAGCAGCTGCTTTTCGCTTGTACTGGGTCTCTCTAGGTAGACCAGATCTGAGCCTGGGAGCTCTCTGGCTATCTGGGGAACCCACTGCTTAAGCCTCAATAAAGCTTGCCTTGAGTGCTTCAAGTAGTGTGTGCCCGTCTGTTGTGTGACTCTGGTAACTAGAGATCCCTCAGACCATTTTGGTAGTGTGGAAAATCTCTAGCAGTGGCGCCCGAACAGGGACTCGAAAGCGAAAGTAAGACCAGAGAAGTTCTCTCGACGCAAGACTCGGCTTGCTGAAGTGCACTCGGCAAGAGGCGAGAGCGGCGGCTGGTGAGTACGCCAAATTTTATTTGACTAGCGGTGGCTAGAAGGAGAGAAGATGGGTGCGAGAGCGTCAATATTAAGAGGGGGAAAATTAGATACATGGGAAAAGATTAGGTTAAGGCCAGGGGGAAAGAAACACTATATGATAAAACATTTAGTATGGGCAAGCAGGGAGCTGGAAAGATTTGCACTTAACCCTGGCCTTTTAGAAACAGCAGATGGCTGTAAACAAATAATAAAACAGCTACACCCAGCTCTTAAGACAGGAACAGAGGAACTAAGGTCATTATACAACACAGTAGCAACCCTCTATTGTGTCCATGCAAAGATAGAGGTAAGAGACACCAAGGAAGCCTTAGACAAGATAGAGGAAGAACAAAACAAAAGTCAGCAAAAAATACAGCAGGCAAAAGAGGATGACAAGAAGATCAGTCAAAATTATCCTATAGTGCAGAATCCTCAAGGGCAAATGGTACACCACCCACTATCGCCTAGAACTTTGAATGCATGGGTGAAAGTAGTAGAGGAGAAGGCTTTTAGCCCAGAGGTAATACCCATGTTTACAGCACTATCAGAAGGAGCTACCCCACATGATTTAAACACCATGTTAAATACAGTGGGGGGACATCAAGCAGCCATGCAAATGTTAAAAGATACCATCAATGAGGAGGCTGCGGAATGGGATAGATTACATCCAGTACATGCAGGGCCTGTTGCACCAGGCCAAATAAGAGAACCAAGGGGAAGTGACATAGCAGGAACAACTAGTAACCTTCAGGAACAAATAACATGGATGACAAGTAACCCACCTATTCCAGTGGGAGACATCTATAAAAGATGGATAATTATGGGATTAAATAAAATAGTAAGAATGTATAGCCCTGTCAGCATTTTGGATATAAAACAAGGGCCAAAGGAACCTTTTAGAGACTATGTAGACCGGTTCTTTAAAACTTTAAGAGCTGAACAAGCTTCACAAGATGTAAAAAATTGGATGACAGACACCTTGTTGGTCCAAAATGCGAACCCAGATTGTAAGATCATTTTAAGAGGATTAGGACCAGGGGCTACATTAGAAGAAATGATGACAGCATGTCAGGGAGTGGGAGGACCTAGCCACAAAGCAAGAGTGTTGGCTGAAGCAATGAGCCAAGCAAACAATATAACCATGATGCAGAGAAGCAATTTTAAAGGCCCTAAAAGAACTGTTAAATGTTTTAACTGTGGCAAGGAAGGACACATAGCCAGAAATTGCAGGGCTCCTAGGAAAAAAGGCTGTTGGAAATGTGGACAGGAAGGACACCAAATGAAAGATTGTACTGAAAGACAGGCTAATTTTTTAGGGAAAATTTGGCCTTCCCACAAGGGGAGGCCAGGGAATTTCCTTCAGAACAGACAAGAGCTGCAGACCAGACTAGAGCCAACAGCCCCACCAGCAGAGAGCTTCAGGTTCGAGGAGACAACCCCCGCCCCGAAGCAGGAGCCGAGGGACAGGGAACCCTTAACTTCCCTCAAATCACTCTTTGGCAACGACCCATTGTTACAATAAAAATAGGGGGCCAAATAAAGGAGGCCCTCTTAGACACAGGAGCAGATGATACAGTATTAGAAGAAATAAATTTGCCAGGAAAATGGAAACCAAAAATGATAGGAGGAATTGGAGGTTTTATCAAAGTAAGACAGTATGATCAAATACTTATAGAAATTTGTGGAAAAAAGGCTATAGGTACAGTGTTAGTGGGACCTACACCAGTCAACATAATTGGAAGGAATATGTTGACTCAACTTGGATGTACACTAAATTTTCCAATTAGTCCCATTGAAACTGTACCAGTAAAATTAAAGCCAGGAATGGATGGCCCAAGGGTTAAACAATGGCCATTGACAGAAGAGAAAATAAAAGCATTAACAGCAATTTGTGAAGAAATGGAAAAGGAAGGAAAAATTACAAAAATAGGGCCTGAAAATCCATATAACACTCCAGTATTTGCTATAAAAAAGAAGGACAGTACTAAATGGAGAAAATTAGTAGATTTCAGGGAACTCAATAAAAGAACTCAAGACTTTTGGGAAGTGCAATTAGGAATACCACACCCAGCAGGGTTAAAGAAGAAAAAATCAGTGACAGTACTAGATGTGGGGGATGCATATTTTTCAGTGCCTTTAGACGAAGGCTTCAGGAAATATACTGCATTCACCATACCTAGTATAAACAATGAAACACCAGGAATTAGATATCAATATAATGTGCTTCCACAGGGATGGAAAGGATCACCAGCAATATTCCAGAGTAGCATGACAAAAATACTAGAACCCTTTAGGGCACAAAATCCAGGAATAGTCATCTATCAATATATGGATGACTTGTATGTAGGATCTGACTTAGAAATAGGACAACATAGAGCAAAGATAGAGGAATTAAGAGAACATCTATTAAAGTGGGGATTTACCACACCAGACAAGAAACATCAGAAAGAACCCCCGTTTCTTTGGATGGGGTATGAGCTCCATCCTGACAAATGGACAGTACAGCCTATAAAGTTACCAGAAAAGGATAGCTGGACTGTCAATGATATCCAGAAGTTAGTGGGAAAATTAAACTGGGCAAGTCAGATTTACCCCGGGATTCAAGTAAAACAACTATGTAAACTCCTTAGGGGGGCCAAAGCACTAACAGACATAATACCACTAACTGAAGAGGCAGAATTAGAATTGGCAGAGAACAGGGAAATTCTAAAAGAACCAGTACATGGAGTATATTATGATCCATCAAAAGACTTGATAGCTGAAATACAGAAACAGGGGCATGATCAATGGACATATCAAATTTACCAGGAACCATTCAAAAATCTAAAAACAGGGAAGTATGCAAAAATGAGGACTGCCCACACTAATGATGTAAAACAGTTAACAGAGGCAGTGCAAAAAATAGCCATAGAAAGCATAGTAATATGGGGAAAAACTCCCAAATTTAGATTACCCATTCAAAAAGAAACATGGGAGACATGGTGGACAGACTATTGGCAAGCCACCTGGATTCCTGATTGGGAGTTTGTTAATACTCCTCCCCTAGTAAAATTATGGTACCAGCTGGAGAAAGAACCAATAGTAGGAGCAGAAACTTTCTATGTAGATGGAGCAGCCAATAGGGAAACTAAGATAGGAAAAGCAGGATATGTTACAGATAGGGGAAGACAGAAGGTTATTTCTCTAACTGAAACAACAAATCAGAAGACTGAATTACATGCAATTCATCTAGCTTTGCAAGATTCAGGATCAGAAGTAAACATAGTAACAGACTCACAGTATGCACTAGGAATCATTCAAGCACAACCAGATAAGAGTGAATCAGAGTTAGTCAACCACATAATAGAACAATTGATAAAAAAGGAAAGGGTCTATCTGTCATGGGTACCAGCACATAAGGGAATTGGAGGAAATGAACAAGTAGATAAACTAGTAAGTAGTGGAATCAGAAAAGTACTATTTTTAGATGGAATAGATAAAGCTCAAGAAGAGCATGAAAAATATCACAGTAATTGGAGAGCAATGGCCAGTGATTTTAATTTGCCACCCATAGTAGCAAAAGAAATAGTAGCTAGCTGTGATAAATGTCAGCAAAAAGGGGAAGCCATACATGGACAAGTAGACTGTAGTCCAGGGATATGGCAGTTAGATTGTACACATTTAGAAGGAAAAGTCATCCTAGTAGCAGTCCATGTGGCCAGTGGCTACATAGAAGCAGAGGTTATCCCAGCAGAGACAGGACAAGAAACAGCATACTATATACTAAAATTAGCATCAAGATGGCCAGTCAAAGTAATACATACAGACAATGGTGGTAATTTCACTAGTGCTGCAGTTAAGGCAGCCTGTTGGTGGGCAGGTATCCAACAGGAATTTGGAATTCCCTACAATCCCCAAAGCCAGGGAGTAGTAGAATCCATGAATAAAGAACTAAAGAAAATTATAGGGCAGGTAAGAGAGCAAGCTGAGCACCTTAAGACAGCAGTACAAATGGCAGTATTCATTCACAATTTTAAAAGAAAAGGGGGGATTGGGGGGTACAGTGCAGGGGAAAGAATAATAGACATAATAGCAACAGACATACAAACAAAAGAATTACAAAAACAAATTATAAAAATTCAAAATTTTCGGGTTTATTACAGAGACAGCAGAGACCCTATTTGGAAAGGACCAGCCAAACTACTCTGGAAAGGTGAAGGGGCAGTAGTCATACAAGATAACAGTGACATAAAGGTAGTACCAAGGAGGAAAGCAAAAATCATTAGGGACTATGGAAAACAGATGGCAGGTGCTGATTGTGTGGCAGGTAGACAGGATGAGGATCAAAACATGGAATAGTTTAGTAAAGCACCATATGTATGTTTCAAAAAGAGCTAAGGGATGGTTTTATAGGCATCACTTTGAAAGCAGAAATCCAAAAGTAAGTTCAGAAGTACACATCCCATTAGGGGAGGCCAGATTAGTAATAAAAACATATTGGGGTTTGCAAACAGGAGAAAGGGATTGGCATTTGGGTAATGGGGTCTCCATAGAATGGAGATTGAGAAGATACAGCACACAGGTAGATCCTGGCCTGGCAGACCAGCTAATTCATATGCATTATTTTGATTGTTTTGCAGACTCTGCCATAAGACAAGCCATATTAGGACATATAGTTATTCCTAGGTGTGACTATCAAGCAGGACATAATAAGGTAGGATCTCTACAATACTTGGCACTGACAGCATTGATAAAACCAAGAAAGAGAAAGCCACCTCTGCCTAGTGTTAGGAAATTAGCAGAAGATAGATGGAACAACCCCCAGAAAACCAGGGGCCGCAGAGGGAACCATACAATGAATGGACATTAGAGTTACTAGAGGAACTCAAGCAGGAAGCTGTTAGACACTTTCCTAGACCATGGCTTCATGGATTAGGACAATATATCTATGAAACCTATGGGGATACTTGGACAGGAGTCGAAATTATAATAAGAATCCTGCAACAACTACTGTTTATTCATTTCAGAATTGGGTGCCAACATAGCAGAATAGGCATTATGCGACAAAGAAGAGCAAGAAATGGAGCCAGTAGATCCTAACTTAGAGCCCTGGAACCATCCAGGAAGTCAGCCTAAAACTGCTTGTAATCCTTGCTATTGTAAACGCTGTAGCTACCATTGTCTAGTTTGCTTTCAGAAAAAAGGCTTAGGCATTTCCTATGGCAGGAAGAAGCGGAGACAGCGACGAAGCGCTCCTCCAAGCAGTGAGGATCATCAAGATCTTATATCAAAGCAGTAAGTATATGTAATGATAGATTTAACAGCATTAGGAGTGGCAGCATTGATAGTAGCACTAATTATAGCAATAGTTGTGTGGACCATAGCATATATAGAATATAGGAAATTGGTAAGACAAAGAAAAATAGATTGCTTAATTAAAAGAATTAGGGAAAGAGCAGAAGACAGTGGCAATGAGAGTGAGGGGGATACTGAGGAATTATCAACACTGGTGGATATGGGGAATCTTAGGCTTTTGGATGTTAATGATTTGTAATGTGGTGGGAAACTTGTGGGTCACAGTTTATTATGGGGTACCTGTGTGGAAAGATGCAAAAACTACTCTATTCTGTGCATCAGATGCTAAAGCATATGAGAAAGAAGTGCATAATGTCTGGGCTACACATGCCTGTGTACCCACAGACCCCAACCCACAAGAAATGCTATTGGGAAATGTAACAGAAAATTTCAACATGTGGGAAAATGACATGGTGGATCAGATGCATGAGGATATAATTAGTTTATGGGACGAAAGCCTAAAGCCATGTGTACAGCTGACCCCACTCTGTGTCACCTTAGACTGTAAACCTGCTAATATTAGTAATAAGAATAACTGTAATAGTACCTGTGCGGATGCCATCAGGAAAGAAATAAAAAATTGCACTTTCAATGTAACCACAGAAATAAAAGATAGGAAAAGAAAAGAGTATGCACTTTTTTATAGACTTGATATAGTACCACTACCACCTGAGGGGGAAAGTAGCAACTCTAGTGATAGCGGCAGCTCTAGTGATAATAGGAATATCAGCAACCCTAGTAACTATAGTGAGTATAGATTAATAAATTGTAATACCTCAGCCATAAAACAAGCCTGTCCAAAGGTCTCTTTTGATCCAATTCCTATATATTATTGCACTCCAGCTGGTTATGCGATTCTAAAGTGTAATGATGAGACATTCAATGGGACAGGACCATGTAAGAATGTCAGCACAGTACAATGTACACATGGAATTAAGCCAGTGGTATCAACACAATTATTGTTAAATGGTAGCCTAACAAAAGAAGTAATAATTAGATCTAAAAATCTGACAGACAATGCCAAAACAATAATAGTAAATCTTAATGTATCTGTACCAATTGTGTGTATAAGACCCGGCAATAATACAAGAAAAAGTATAAGGATAGGACCAGGACAAGTATTCTTTACAGCCACAGACATAATAGGAAATATAAGAGAAGCACATTGTAACATTAGTGAGAAAGCATGGAATGAAACCTTACAAAGGGTAGTTAAAAAATTAAAAGAACACTTCCCTAATAAAACAAGAATAAAATTTGACAAACATTCAGGAGGGGATCCAGAAATTACAGCACATAGCTTTAATTGTGGAGGAGAATTTTTCTATTGTGATACATCAAAACTGTTTAATAGGACATACACGGTTAATGACACACACGAGACTAATAGTACTGATACAAACAGTACCATCACACTCCCATGCAGAATAAAACAAATTATAAACATGTGGCAGAGAGTAGGACAAGCAATGTATGCTTCTCCCATTGCAGGAAACATAACATGTAACTCAAGTATCACAGGATTGCTACTGACGAGTGATGAAGGAGGAAATAACAACGAAAACGAGACATTCAGGCCTGGAGGAGGAGATATGAGGGACAATTGGAGAAGTGAATTATATAAATATAAAGTGGTAGAAGTTAAGCCATTGGGAATAGCACCCACCAAGGCAAAAAGGAGAGTGGTGGAGAGAGAGAAAAGAGCAGTAGGAATAGGAGCTTTGTTCCTTGGGTTCTTGGGAGCAGCAGGAAGCACTATGGGCGCGGCGTCAATGACGCTGACGGTACAGGCCAGACAATTGTTGTCTGGTATAGTGCAACAGCAAAGCAATTTGCTGAGGGCTATAGAGGCGCAACAACATATGTTGCAACTCACAGTCTGGGGCATTAAGCAGCTCCAGGCAAGAGTCCTGGCCCTAGAAAGATACCTAAAGGATCAACAGCTCCTAGGGATTTGGGGCTGCTCTGGAAAACTCATCTGCACCACTACTGTGCCTTGGAACTCCAGTTGGAGTAATAGATCTGAAGCAGAAATTTGGGACAACATGACCTGGATGCAGTGGGATAGAGAAATTAGTAATTACACATACACAATATACAACTTGCTTGAACAATCACAAAACCAGCAGGAGAAAAATGAACAGGATTTATTAGCATTAAACAGTTGGCAAAATCTGTGGACTTGGTTTGACATAACAAAGTGGCTGTGGTATATAAAAATATTCATAATGATAGTAGGAGGCTTGATAGGTTTGAGAATAACTTTTGCTTTGCTTTCTATAGTGAATAGAGTTAGGCAGGGATACTCACCCTTGTCGTTGCAGACCCTTACCCCGAACCCAGGGGGACCCGACAGGCACAGAGGAATCGAAGAAGAAGGTGGAGAGCAAGACAGAGACAGATCCATTCGATTAGTGAACGGATTCTTAGCGATCTTCTGGGACGACCTGCGGAGCCTGTGCCTTTTCATCTACCACCGATTGAGGGACTTCATATTAGTGACAGCGAGAGCGGTGGAACTTCTGGGACGCGGGGGGTGGGAAGCCCTTAAGTATCTGGGAGGCTTTGTGCAGTATTGGGGTCTGGAACTGAAAAAGAGTGCTATTAGTCTGCTTGATACCATAGCAATAACAGTAGCTGAAGGAACAGATAGAATTATAGAAATAGTACAAAGAATTTTTAGAGCTATCAGAAACATACCTAGAAGAATAAGACAGGGCTTTGAAACAGCTTTGCTATAAAATGGGGGGCAAGTGGTCAAAAAGCAAGAGGAGTGACTGGCCAGTTGTGAGAGAAAGAATAAGACAAACTGAGCCAGCAGCAGAGGGAGTAGGGGCAGCTTCTCAAGACTTAGGTAAGCATGGAGCAATTACAAGCAGCAACACAGACACCAATAATGCTGATTGTGCCTGGCTGCGAGCACAAGAGGAGGAAGATGATGTAGGCTTTCCAGTCAGACCTCAGGTGCCTTTAAGGCCAATGACTTATAAGGAAGCGTTCGATCTCAGCTTCTTTTTAAAAGAAAAGGGGGGACTGGAAGGGTTAATTTACTCCAAGAAGAGGCAAGAAATCCTTGATCTGTGGGTTTATCACACACAAGGCTTCTTCCCTGATTGGCAAAACTACACACCAGGACCAGGGGTCAGATACCCACTGACCTTTGGATGGTGCTACAAGCTAGTGCCAGTAGACCCAAGTGAAGTGGAAGAAGCCAGCGGAGGAGAAGACAACTGCCTGTTACACCCTATGAACCTGCATGGAATAGAGGATGAACACAGAGAAGTATTAAAATGGAAGTTTGACAGTCAACTAGCACGCAGACACCTGGCCCGTGAGAAACATCCGGAGTTTTACAAAGACTGCTGACACAGAAAGGACTTTGCTGACACAGAAGGGACTTTCCGCTGGGACTTTCCACTGGGGCGTTCCAGGGGGAGTGGTCTGGGCGGGACGGGGAGTGGTCAACCCTCAGATGCTGCATATAAGCAGCTGCTTTTCGCTTGTACTGGGTCTCTCTAGGTAGACCAGATCTGAGCCTGGGAGCTCTCTGGCTATCTGGGGAACCCACTGCTTAAGCCTCAATAAAGCTTGCCTTGAGTGCTTCAAGTAGTGTGTGCCCGTCTGTTGTGTGACTCTGGTAACTAGAGATCCCTCAGACCATTTTGGTAGTGTGGAAAATCTCTAGCA

>CH256

TGGAAGGGTTAATTTACTCCAAGAAAAGGCAAGACATCCTTGATTTGTGGGTTTATAACACACAAGGCTTCTTTCCTGATTGGCAAAACTACACACCGGGACCAGGGGTCAGATATCCACTGACCTTTGGATGGTGCTTCAAGCTAGTACCAGTTGACCCAAGGGAAGTAGAAGAAGCCAACGAAGGAGAAAACAACTGTCTGCTACACCCTATGAGCCAACATGGAATGGATGATGATCACAGAGAAGTATTAAAGTGGAAGTTTGACAGTCAACTAGCACACAAACACATGGCCCGCGAGCTACATCCGGAGTTTTACAAAGACTGCTGACACAAAAGGGACTTTCCGCTGGGACTTTCCACTTGGGCGTTCCAGGAGGTGGGGTCTGGGCGGGACCGGGGAGTGGCCAGCCCTCAGATGCTGCATATAAGCAGCTGCTTTTCGCCTGTACTGGGTCTCTCTAGGTAGACCAGATCTGAGCCTGGGAGCTCTCTGGCTGCCTAGGGAACCCACTGCTTAAGCCTCAATAAAGCTTGCCTTGAGTGCTCTAAGTAGTGTGTGCCCGTCTGTTGTGTGACTCTGGTAACTAGAGATCCCTCAGACCATTTTGGTTGCGTGGAAAATCTCTAGCAGTGGCGCCCGAACAGGGACTTGAAAGCGAAAGTAAGACCAGAGGAGATCTCTCGACGCAGGACTCGGCTTGCTGAAGTGCACTCGGCAAGAGGCGAGAGCGGCGACTGGTGAGTACGCCAATTTTATTTTGACTAGCGGAGGCTAGAAGGAGAGAGATGGGTGCGAGAGCGTCAATATTAAGAGGGGGAAAATTAGATAAATGGGAAAAAATTAGGTTAAGGCCAGGGGGAAAGAAAAAATATCAGCTAAAACACCTAGTATGGGCAAGCAGGGAGCTGGAAAAATTTGCACTTAACCCTGACCTTTTAGAAACATCAGAAGGCTGTAAACAAATAATAAGACAGCTACACCCAGCTCTCCAGACAGGAACAGAGGAACTCAAATCACTATATAACACAGTAGCAACTCTCTATTGTGTACATAAAAATATAGATGTGCGAGACACCAAGGAGGCCTTAGACAGGATAGAGGAGGAACAAAACAAATGTCAGCAAAAAACACAGCAGGCAGCAGCGGCTGACGGACAGGTCAGTCGAAATTTTCCTATAGTACAGAATCTTCAAGGGCAAATGGTACACCAGCCCTTATCACCTAGAACTTTGAATGCTTGGGTAAAAGTAGTAGAGGAGAAGGGTTTCAACCCAGAGGTAATACCCATGTTTACAGCATTATCAGAAGGAGCCACCCCACAAGATATGAACACTATGTTAAATACAGTGGGGGGACATCAAGCAGCCATGCAAATGTTAAAAGATACCATCAATGAGGAGGCTGCAGAGTGGGATAGATTACATCCAGTACAGGCAGGGCCTGTTGCACCAGGCCAAATAAGGGAACCAAGGGGAAGTGACATAGCAGGAACTACTAGTACCCTTCAGGAACAAATAGCATGGATGACAAGTAACCCACCTGTTCCAGTGGGAGACATCTATAAAAGATGGATAATTCTGGGGTTAAATAAAATAGTAAGAATGTATAGCCCTGTCAGCATTTTGGACATAAAACAAGGACCAAAGGAACCCTTTAGAGACTATGTAGATCGGTTCTTTAAAACTTTAAGGGCTGAACAGGCTACACAAGATGTAAAAAATTGGATGACAGACACCCTGTTGGTCCAAAATGCGAACCCAGATTGCAAGACCATTTTAAGAGCACTAGGACCAGGGGCTACATTAGAGGAAATGATGACAGCATGTCAAGGAGTGGGAGGACCTAGCCACAAAGCAAGAGTGTTAGCCGAGGCAATGAGCCAAACAAACACAAACATACTGATGCAGAGAAGCAATTTCAAAGGCACTAGAAGAATTGTTAAATGTTTCAACTGTGGCAAGGAAGGGCACATAGCCAAAAATTGCAGGGCCCCTAGGAAAAAGGGCTGTTGGAAATGTGGAAAGGAAGGACACCAAATGAAAGACTGTACAGAAAGGCAGGCTAATTTTTTAGGGAAAATTTGGCCTTCCCACAAGGGGAGGCCAGGGAATTTCCTTCAGAACAGGCCAGAGCCAACAGCCCCACCACTAGAACCAACAGCCCCACCAGCAGAGAGCTCCACCCCCGCTCTGAAGCAGGAGCAGAAAGACAGGGAACCCTCAATTTCCCTCAAATCACTCTTTGGCAACGACCCCTTGTCACAATAAGAGTAGGGGGACAAATAAAGGAGGCTCTATTAGACACAGGAGCAGATGACACAGTATTAGAAGAATTAAGTTTGCCAGGAAAATGGAAACCAAAAATGATAGGAGGAATTGGAGGATTTATCAAAGTAAGACAGTATGATCAAATATCTATAGAAATTTGTGGAAAAAAGGCTATAGGTACAGTATTGGTAGGACCTACACCTGTCAACATAATTGGAAGAAACATGTTGACTCAGCTTGGATGCACCCTAAATTTTCCAATTAGTCCCATTGAAACTGTACCAGTAAAATTAAAGCCAGGAATGGATGGCCCAAAGGTTAAACAATGGCCATTGACAGAAGAAAAAATAAAAGCATTAACAGCAATTTGTGAAGAAATGGAAAAGGAAGGAAAAATTACAAAAATTGGGCCTGAAAACCCATATAACACTCCAGTATTTGCCATTAAAAAGAAGGACAGTACAAAGTGGAGAAAATTAGTAGATTTCAGGGAACTTAATAAAAGGACTCAAGACTTTTGGGAAGTTCAATTAGGAATACCACACCCAGCAGGGTTAAAAAAGAAAAAATCAGTGACAGTACTGGATGTGGGGGATGCATATTTTTCAGTTCCTTTAGATAAAGATTTCAGGAAATATACTGCATTCACCATACCTAGTATAAACAATGAAACACCAGGAATTAGATATCAATATAATGTGCTTCCACAAGGATGGAAAGGATCACCAGCAATATTCCAGAGTAGCATGACAAAAATCTTAGAGCCCTTTAGAGCAAAAAACCCAGAAATAGTCATCTATCAATATATGGATGACTTATATGTAGGGTCTGACTTAGAAATAGGGCAACATAGAGCAAAAATAGAGGAGTTAAGAAAACACCTATTGAAATGGGGATTTACCACACCAGACAAGAAACATCAGAAGGAGCCCCCATTTCTTTGGATGGGGTATGAACTCCATCCTGACAAATGGACAGTACAGTCTATACAGCTACCGGAAAAGGACAGCTGGACTGTCAATGATATACAGAAGTTAGTGGGAAAATTAAACTGGGCAAGTCAGATTTACCCAGGAATTAAAGTAAAGCAAATGTGTAAACTCCTTAGGGGAGCCAAAGCACTAACAGATGTAGTACCACTGACTGAAGAAGCAGAATTAGAATTGGCAGAGAACAGGGAAATTCTAAAAGAACCAGTACATGGAGTATATTATGACCCATCAAAAGACTTAATAGCAGAAATACAGAAACAGGGAGATGACCAGTGGACATATCAAATTTACCAAGAACCATTCAAAAATCTAAAAACAGGAAAATATGCAAAACAGAGAACTGCCCACACAAATGATGTAAAACAATTAACTGAGGCAGTGCAGAAAATATGCATGGAAAGCATAGTAATATGGGGGAAGATTCCTAAGTTTAGACTACCCATCCAAAAGGAAACATGGGAGACATGGTGGACAGACTACTGGCAAGCCACCTGGATTCCTGAGTGGGAATTTATTAATACCCCTCCCCTAGTAAAATTATGGTATCAGCTGGAGAAGGAACCCATAGCAGGGGCAGAAACTTTCTATGTAGATGGAGCAGCTAATAGGGAAACTAAAAAGGGAAAAGCAGGTTATGTTACTGACAGAGGAAGGCAGAAAGTTGTTTCTCTAACTGGAACAACAAATCAAAAGACTGAGTTACAAGCAATTCAGCTAGCTTTGCAAGACTCAGGATCAGAAGTAAATATAGTAACAGACTCACAGTATGCATTAGGAATCATTCAAGCACAACCAGATAAGAGTGAATCAGAGTTAGTCAATCAAATAATAGAACAGCTAATAAAAAAGGAAAGAATCTACCTGTCATGGGTACCAGCACACAAAGGAATTGGAGGAAATGAACAAGTAGATAAATTAGTAAGTAGTGGAATCAGGAAAGTACTGTTTCTAGATGGAATAGATAAAGCTCAAGAAGACCATGAAAGATATCACAGCAATTGGAGAGCGATGGCTAGCGAGTTTAATCTGCCACCCATAGTAGCAAAAGAAATAGTAGCCAGCTGTGATAAATGTCAGCTAAAAGGGGAAGCCATACATGGACAAGTAGACTGTAGTCCAGGGGTATGGCAACTAGATTGTACACACTTAGAAGGGAAAATCATCCTGGTAGCAGTCCATGTAGCCAGTGGATACATAGAAGCAGAAGTCATCCCAACAGAAACAGGACAAGAAACAGCATACTATATACTAAAATTAGCAGGAAGATGGCCAGTCAAAGTAATACATACAGACAATGGCCCTAATTTTACCAGTGCTGCAGTTAAGGCAGCCTGTTGGTGGGCAGGTATCCAACAGGAATTTGGAATTCCCTACAATCCCCAAAGTCAGGGAGTAGTGGAATCCATGAATAAAGAATTAAAGAAAATCATAGGGCAAGTAAGAGATCAAGCTGAGCACCTTAAGACAGCAGTACAAATGGCAGTGTTCATTCACAATTTTAAAAGAAAAGGGGGGATTGGGGGGTACACTGCAGGGGAAAGAATAATAGACATAATAGCAACAGACATACAAACTAAGCAATTACAAAAACAAATTATAAAAATTCAAAATTTTCGGGTTTATTACAGAGACAGCAGAGATCCAATTTGGAAAGGACCAGCCAAACTACTCTGGAAAGGTGAAGGGGCAGTAGTAATACAAGATAACAGTGACATAAAGGTAGTACCAAGAAGGAAAGCAAAGATTATTAAGGACTATGGAAAACAGATGGCAGGTGCTGATTGTGTGGCAGGTAGACAGGATGAAGATTAGAACATGGAATAGTCTAGTAAAGCACCATATGTATGTTTCAAGGAGAGCTGATGGATGGTTTTACAGACATCACTATGAAAGCAGACATCCAAAAATAAGTTCAGAAGTACACATCCCATTAGGGGATGCTAGATTAGTAATAAAAACATATTGGGGTTTGCAGACAGGAGAAAGAGCTTGGCATTTGGGTCATGGAGTCTCCATAGAATGGAGACTGAAAAGATATAGCACACAAGTAGACCCTGACCTGGCAGATCAACTAATTCATATGCATTATTTTGATTGTTTTGCAGACTCTGCCATAAGACAAGCCATACTAGGACACGTAGTTAGCCCTAGGTGTGACTATCAAGCAGGACATAACAAGGTAGGATCTCTACAATACTTGGCACTGACAGCATTGATAAAGCCAAAAAAGATAAAGCCACCTCTGCCTAGTGTTAGCAAGTTAGTAGAGGATAAATGGAACAAGCCCCAGAAGACCAGGGGCCGCAGAGGGAACCATACAATGAATGGACACTAGAGCTTCTAGAGGAACTCAAGCAGGAAGCTGTCAGACACTTTCCTAGGCCATGGCTTCACAGTTTAGGACAACATATCTATGAAACCTATGGGGACACTTGGACGGGAGTTGAAGCCATAATAAGAATTCTGCAACAACTGTTGTTTGTTCATTTCAGAATTGGGTGCCAGCATAGCAGAATAGGCATTATACGACAGAGAAGAGCAAGAAATGGAGCCAATAGATCCTAAACTAGAGCCCTGGAATCATCCAGGAAGCCAGCCTAAGACTGCTTGTAATAAGTGCTATTGTAAAAAATGTAGCTATCATTGTCTAGTTTGCTTTCAGACAAAAGGCTTAGGCATTTCCTATGGCAGGAAGAAGCGGAGACAGCGACGAAGCGCTCCTCCAAGCAGTAAGGATCATCAAAATCCTCTACCAAAGCAGTAAGTATATGTAATGTTAGACTTAGTAGCAAGAGTAGATTATAGAATAGGAGTAGGAGCATTAATAGTAGCACTAATCATAGCAATAGTTGTGTGGATCCTAGCATATATAGAATATAAAAGGTGGTTAAAACAAAAGAAAATAGACTGGTTAATTAAAAGAATTAGGGAAAGAGCAGAAGACAGTGGCAATGAGAGTGAGGGGGATACAGAGGAATTATCAACAATGGTGGATATGGAGCATCTTGGCCTTTTGGATGTTAATGGTTAGTGTGAGGGGAAACTTGTGGGTTACAGTCTATTATGGGGTACCTGTGTGGAAAGAAGCAAAAACTACTCTATTCTGTGCATCAGATGCTAAAGCATATGACAAGGAAGTGCATAATGTCTGGGCTACACATGCCTGTGTACCCACAGACCCCAACCCACAAGAAATAAAATTGGAAAATGTAACAGAAAATTTTAATATGTGGAAAAATGATATGGTGGATCAGATGCATGAGGATATAATCAGTTTATGGGATCAAAGCCTAAAGCCATGTGTGAAGTTGACCCCACTTTGTGTCACTTTAAACTGTAGTCATAATATTACGGTTAATGGTACCATGGGAAATGGTACCAGGGGAAATGATAGTACGATTGGGAAAATGGGAGATGAAATGACAAATTGCTCTTTCAATGCAACCACAGAAATAAAAGATAAGAAAAAGCTAGAATATGCACTTTTTTATAAACTTGATGTAGTAACACTTGAGGAAAACTCCAGTGAGTACAGATTAATAAATTGTAATACCTCAGTAGTTACACAAGCCTGTCCAAAGGTTTCGTTTCACCCAATTCCTATACATTATTGTGCTCCTGCTGGTTATGCGATTCTAAAGTGTAATAATAAGACATTCAATGGAACAGGACCATGCAATAAGATCAGCACTGTACAGTGTACACATGGGATCAAGCCAGTGGTATCTACTCAACTACTGTTAAATGGTAGTCTAGCAGAAAAAGAGATAATAGTTAGATCTGAAAATATACAGGACAATGTCAAAACAATCATAGTACATCTTAATGAATCTGTGAAAATTGAGTGTACAAGGCCCGGCAATAATACAAGAAAAAGTGTGAGGATAGGACCAGGACAAACATTCTTTGCAACAGGAGACATAATAGGAGATATAAGACAAGCATACTGTACCATTAATGGGACTCAATGGAATGACACTTTAAATAGGGTAAGAGAAACATTACAAAGGCACTTCCCTAATAAAACAATAAAATTTAGACCACACTCAGGAGGGGACTTAGAAATTACAACACATAGCTTTAATTGTAGAGGAGAGTTTTTCTATTGCAATACATCAAAAATATTTGATAGTGACAATGCAAACAAAACTTCAGGAAATATCACACTCCCATGCAAAATAAAACAAATCATAAACATGTGGCAGGGGGTAGGAAGAGCAATGTATGCCCCTCCCATTGCAGGAAACATAACATGTACATCAAATATCACAGGACTACTATTGACACGTGATGGAGGCAATGTTACAAATGACACAGAGATATTCAGACCTGGAGGAGGAAATATGAGGGACAATTGGAGAAGTGAATTATACAAATATAAAGTGGTAGAAATTAAGCCATTAGGGATAGCACCCACCAAGGCAAAAAGGCGAGTGGTGGAGAGAGAAAAAAGAGCAGTGGGAATAGGAGCTGTATTCCTTGGGTTCTTGGGAGCAGCAGGAAGCACTATGGGCGCAGCGTCAATAGCGCTGACGGTACAGGCCAGACAATTGTTGTCTGGTATAGTGCAACAGCAAAACAATTTGCTGAGAGCTATAGAGGCGCAACAGCATATGTTGCAACTCACGGTCTGGGGCATTAAACAGCTCCAGGCAAGAGTCCTGGCTATAGAAAGATACCTAGAGGATCAACAGCTCCTAGGGATTTGGGGCTGCTCTGGAAAACTCATCTGCACCACTGCCGTGCCTTGGAACAATAGTTGGAGTAATAAATCTCAAGATGATATTTGGGGCAATATGACCTGGATGCAATGGGACAGAGAAATTAGCAATTACACAGACACAATATACAGGTTGCTTGAAGACTCGCAAATCCAGCAGGAAAAGAATGAAAAGGATTTACTAGCATTGGACAGTTGGCAAAATTTGTGGAATTGGTTTAACATAACAAAATGGCTGTGGTATATAAAAATATTCATAATGATAGTAGGAGGCTTAATAGGTTTAAGAATAATTTTTGCTGTACTATCTATAGTAAATAGAGTTAGGCAGGGATACTCACCTTTGTCGTTTCAGACCCTTATCCCAAACCAGAGGGAACCCGACAGGCTCGGAAGAATCGAAGAAGAAGGTGGAGAGCCAGACAGAAACAGATCCATTCGATTGGTGAACGGATTCTTAGCTCTTACCTGGGACGACCTGCGGAGCCTGTGCCTCTTCAGCTACCACCGATTGAGAGACTTCATATTGGTGACAGTGAGAGTGGTGGAACTTCTGGGACGCAGCAGCTTCAGGGGACTACAGAGGGGGTGGGAAGCCCTTAAGTATCTGGGAAGCCTTGTGCAGTATTGGGGTCTAGAGCTAAAAAAGAGTGCTATTAGTTTGCTTGATACCACAGCAATAGCAGTAGCTGAAGGAACAGATAGGATTATAGAATTCCTACAGAGAATTTGTAGAGCTATCTACCATATACCTAGAAGAATAAGACAGGGCTTTGAAGCAGCTTTGCAATAAAATGGGGGGCAAGTGGTCAAAAAGCAGTATAGTTGGATGGCCTAATGTAAGGGAAAGAATAAGGCGAACTGATCCAGCAGCAGAGGGAGTAGGAGCAGCATCTCGAGACTTAGAGAGACATGGGGCACTTACTACCAGCAACACAGTCAGCAACAATGCTGCATGTGCCTGGCTGGAAGCACAAGAAGAGGAAAAAGAAGTAGGCTTTCCAGTCAGACCTCAAGTGCCTTTAAGACCAATGACTTATAAAGGAGCATTCGATCTCAGCTTCTTTTTAAAAGAAAAGGGGGGACTGGAAGGGTTAATTTACTCCAAGAAAAGGCAAGACATCCTTGATTTGTGGGTTTATAACACACAAGGCTTCTTTCCTGATTGGCAAAACTACACACCGGGACCAGGGGTCAGATATCCACTGACCTTTGGATGGTGCTTCAAGCTAGTACCAGTTGACCCAAGGGAAGTAGAAGAAGCCAACGAAGGAGAAAACAACTGTCTGCTACACCCTATGAGCCAACATGGAATGGATGATGATCACAGAGAAGTATTAAAGTGGAAGTTTGACAGTCAACTAGCACACAAACACATGGCCCGCGAGCTACATCCGGAGTTTTACAAAGACTGCTGACACAAAAGGGACTTTCCGCTGGGACTTTCCACTTGGGCGTTCCAGGAGGTGGGGTCTGGGCGGGACCGGGGAGTGGCCAGCCCTCAGATGCTGCATATAAGCAGCTGCTTTTCGCCTGTACTGGGTCTCTCTAGGTAGACCAGATCTGAGCCTGGGAGCTCTCTGGCTGCCTAGGGAACCCACTGCTTAAGCCTCAATAAAGCTTGCCTTGAGTGCTCTAAGTAGTGTGTGCCCGTCTGTTGTGTGACTCTGGTAACTAGAGATCCCTCAGACCATTTTGGTTGCGTGGAAAATCTCTAGCA
